# Systems-level metabolism of the altered Schaedler flora, a complete gut microbiota

**DOI:** 10.1038/ismej.2016.130

**Published:** 2016-11-08

**Authors:** Matthew B Biggs, Gregory L Medlock, Thomas J Moutinho, Hannah J Lees, Jonathan R Swann, Glynis L Kolling, Jason A Papin

**Affiliations:** 1 Department of Biomedical Engineering, University of Virginia, Charlottesville, VA, USA; 2 Division of Computational and Systems Medicine, Department of Surgery and Cancer, Imperial College, London, UK; 3 Division of Infectious Diseases and International Health, Department of Medicine, University of Virginia, Charlottesville, VA, USA

## Abstract

The altered Schaedler flora (ASF) is a model microbial community with both *in vivo* and *in vitro* relevance. Here we provide the first characterization of the ASF community *in vitro*, independent of a murine host. We compared the functional genetic content of the ASF to wild murine metagenomes and found that the ASF functionally represents wild microbiomes better than random consortia of similar taxonomic composition. We developed a chemically defined medium that supported growth of seven of the eight ASF members. To elucidate the metabolic capabilities of these ASF species—including potential for interactions such as cross-feeding—we performed a spent media screen and analyzed the results through dynamic growth measurements and non-targeted metabolic profiling. We found that cross-feeding is relatively rare (32 of 3570 possible cases), but is enriched between *Clostridium* ASF356 and *Parabacteroides* ASF519. We identified many cases of emergent metabolism (856 of 3570 possible cases). These data will inform efforts to understand ASF dynamics and spatial distribution *in vivo*, to design pre- and probiotics that modulate relative abundances of ASF members, and will be essential for validating computational models of ASF metabolism. Well-characterized, experimentally tractable microbial communities enable research that can translate into more effective microbiome-targeted therapies to improve human health.

## Introduction

The microbiome is enormously complex and its composition varies not only between individuals, but within the same individual spatially and temporally (Lozupone *et al.*, 2012; Faith *et al.*, 2013). Most of the microorganisms that comprise the microbiome variously interact through forms of competition and cooperation that are largely uncategorized (Ji and Nielsen, 2015). Despite the daunting complexity of this system, a great deal of research effort is expended with the goal of identifying governing principles that will allow prevention and treatment of a range of human conditions connecting the immune system (Gevers *et al.*, 2014), diet and metabolism (Wang *et al.*, 2015), emotional health (Steenbergen *et al.*, 2015) and other relevant systems (Cho and Blaser, 2012) to the microbiome. If sufficiently understood, there is enormous therapeutic potential in microbiome modulation.

It is well-established that the composition of microbial communities is linked to host health, but many studies linking the microbiome to health-related outcomes provide descriptive or correlative results rather than establish causation (Dodd *et al.*, 2015; Ji and Nielsen, 2015). In addition, despite growing databases of reference genomes, many species detected in these studies are new, if they are detected at all (Nielsen *et al.*, 2014). Germ-free animals colonized specifically with known microorganisms—gnotobiotic animals—enable experiments that can establish causation (Faith *et al.*, 2014). Such experiments cannot easily be performed in humans, making gnotobiotic animals crucial to studying microbiome structure and function.

Germ-free and gnotobiotic mice often do not develop normal immune systems or gastrointestinal function (Brestoff and Artis, 2013). This problem was addressed in part by work in which a cocktail of eight microbial species known as the altered Schaedler flora (ASF) was identified (Schaedler *et al.*, 1965; Dewhirst *et al.*, 1999). Germ-free mice colonized exclusively with the ASF develop relatively normal immune systems and gastrointestinal function (Geuking *et al.*, 2011; Wymore Brand *et al.*, 2015). ASF-colonized mice are commercially available and widely used (Singer and Nash, 2000; Sarma-Rupavtarm *et al.*, 2004; Ge *et al.*, 2006; Stehr *et al.*, 2009; Collins *et al.*, 2014; Henderson *et al.*, 2015; Moghadamrad *et al.*, 2015; Shen *et al.*, 2015). The ASF serves as an experimentally tractable surrogate for wild-type microbiomes.

A limiting factor in ASF-based research to-date is the paucity of knowledge about the eight species contained in the ASF. Little is known about the genetics, metabolism, or *in vitro* characteristics of these eight species, because their primary value historically has been to standardize mice (Dewhirst *et al.*, 1999). Draft genome sequences for all eight ASF member species were published recently (Wannemuehler *et al.*, 2014). Future efforts to understand the mechanistic underpinnings of ASF-host interactions, or ASF dynamics within the host, will depend on a much deeper knowledge of the physiology and metabolism of each ASF member individually, and the interactions among them. To facilitate this goal, we exhaustively compared the functional gene content of all ASF species among each other, to wild-type murine metagenomes, and to random consortia of similar taxonomic composition. We developed a chemically defined medium and performed the first *in vitro* analysis of the growth and metabolism of ASF member species. Finally, we experimentally determined the effects on growth and metabolism of spent media interactions between members of the ASF. The results of this study will serve as a resource for future ASF-based research, and provide a strong foundation for future computational modeling efforts. By better understanding the ASF—including interactions between its members—it will be possible to glean more from ASF-based mouse experiments, thus increasing the value of ASF-colonized mice as a model system for microbiome-host interactions.

## Materials and methods

### Strain information

All strains are identified by the associated ASF number. We performed experiments with ASF356 (*Clostridium* sp.), ASF360 (*Lactobacillus intestinalis*), ASF361 (*Lactobacillus murinus*), ASF457 (*Mucispirillum schaedleri*), ASF492 (*Eubacterium plexicaudatum*), ASF500 (*Pseudoflavonifractor* sp.), *ASF502* (*Clostridium* sp.) and ASF519 (*Parabacteroides goldsteinii*; Wymore Brand *et al.*, 2015). All strains were grown in an anaerobic chamber (Shel Lab BactronEZ, Cornelius, OR, USA) with mixed anaerobic gas (5% carbon dioxide, 5% hydrogen, 90% nitrogen) at 37 °C. Anaerobic conditions were confirmed periodically using an anaerobic indicator (Oxoid, Basingstoke, UK). All strains were propagated on supplemented Brain–Heart Infusion agar.

### Media preparation

Supplemented Brain–Heart Infusion medium: Brain–Heart Infusion base (BD, Franklin Lakes, NJ, USA) was supplemented with yeast extract (5 g l^−1^), vitamin (2 μ l^−1^), hemin (5 mg l^−1^), cysteine (0.5 g l^−1^) and 5% each of newborn calf serum, horse serum, and sheep serum.

Supplemented LB medium: LB base in powder form (Sigma, St Louis, MO, USA) was combined with l-cysteine (Sigma), added KH_2_PO_4_ (6 g l^−1^), (NH_4_)_2_SO_4_ (6 g l^−1^), NaCl (12 g l^−1^), MgSO_4_·7H_2_O (2.5 g l^−1^), CaCl_2_·2H_2_O (1.6 g l^−1^), l-cysteine (0.25 g l^−1^) (see detailed formulation in the Supplementary Materials) and deionized water, which was autoclaved at 121 °C for 20 min. After cooling, vitamin K1 (9.84 mg l^−1^) and filter sterilized (0.22 μm pore size) solutions of hemin (0.005 g l^−1^), lactose (0.05 g l^−1^) and Tween-20 (0.01 g l^−1^) were added.

All media was equilibrated overnight in the anaerobic chamber before inoculation with ASF members.

### Genomic analysis and comparison with wild murine microbiota

Shotgun sequencing metagenomic data from the feces of 15 wild mice from a previous study (Wang *et al.*, 2014) were used as a reference data set (Shannon diversity of 163±72) for comparative analysis to the ASF. We downloaded protein sequences for all 15 samples, which were then annotated with HMMER Version 3.1b2 (Eddy, 1998), using bactNOG (144 498 protein sequences) from eggNOG version 4.1 (Powell *et al.*, 2014) as the profile hidden Markov models. For each gene call, a non-supervised orthologous group (NOG) was assigned using the database target with the lowest *E*-value below 10^−10^. Overall, 22.3% of metagenomic open reading frames were assigned a NOG annotation. Protein sequences for each ASF species were downloaded from GenBank and annotated using the same procedure.

To compare metagenome coverage by the ASF to coverage by random communities, species were drawn from among the 989 Firmicutes and 176 Bacteroidetes in bactNOG in a 6:2 ratio, respectively, to represent the most abundant phyla in the mouse gastrointestinal tract (Nguyen *et al.*, 2015). The complete list of Firmicutes and Bacteroidetes in bactNOG version 4.1 is available in Supplementary Data 1. Random communities of size 8, 16 and 32 were compared with the ASF for percent coverage of NOGs annotated in any metagenomic sample. This coverage was further sorted by sample frequency, where each NOG can occur in up to 15 metagenomic samples. NOGs containing functional annotations in more than one category were discarded during all portions of analysis (representing <1% of total annotations in any sample).

### Preparation of spent media

Spent media from each ASF member was prepared by growing each species in supplemented LB for 70 h. The resulting culture was centrifuged at 3500 r.p.m. for 10 min and the supernatant was filter sterilized (PVDF membranes with 0.22 μm pore size). Aliquots of spent medium were stored at −80 °C. Individual aliquots were thawed and equilibrated in the anaerobic chamber overnight before inoculation.

### Growth measurements

Growth curves were obtained for ASF members in the anaerobic chamber using four miniaturized plate readers measuring optical density at 870 nm (Jensen *et al.*, 2015). Overnight liquid cultures of 10 ml were prepared for each ASF member: The entire volume of the overnight cultures were centrifuged at 8000 r.p.m. for 2 min and the resulting pellets were resuspended in fresh liquid medium to produce a dense suspension of 0.75 ml. The optical density of the suspension was obtained on a Tecan (Männedorf, Switzerland) plate reader at 600 nm. Liquid cultures were prepared in six-well plates with 6 ml per well, and inoculated (from the dense suspension) to a starting OD600 of 0.001. Each experimental condition was replicated four times. Each plate was covered with a Breath-Easy membrane (Sigma). The OD870 was tracked for 70 h. At the final time point, the OD600 was measured for each well on the Tecan. The growth curves obtained at OD870 were normalized to the initial and final OD600 measurements (Supplementary Materials and Methods). For each well of the six-well plate, growth curves from four independent LED pairs were averaged to produce a single growth curve per well. To determine the area under a growth curve, we applied trapezoidal numerical integration. The R (The R Foundation, Vienna, Austria) code for growth curve analysis is available in an open online repository (see Code and data availability).

### Determining substrate utilization and by-product consumption with NMR spectroscopy

Media (fresh or spent) samples of 2 ml were filter sterilized (0.22 μm pore size) and frozen at −80 °C. Standard one-dimensional (1D) ^1^H-NMR spectra with water pre-saturation were acquired at 300 K using a 600 MHz Avance III spectrometer (Bruker, Rheinstetten, Germany). Spectra were imported into Matlab R2014a (The Mathworks, Inc., Natick, MA, USA). Biologically irrelevant regions of the spectra were removed (TSP resonance at δ^1^H 0 and residual water peak δ^1^H 4.5–5.2) before peak alignment by recursive segment-wise peak alignment (Veselkov *et al.*, 2009). The loadings of pairwise principal component analysis models, comparing blank media with the spent media of each bacteria species, were used to identify metabolites generated or consumed in each experiment. The relevant regions of the spectra were integrated to calculate relative spectral intensities for each metabolite. Relative intensities in spent and double spent media were converted to z-scores with respect to metabolite abundances in fresh media. We defined significant abundance changes as those of magnitude greater than ±2 standard deviations from zero (zero being the metabolite abundance in fresh media). The peak integral data and associated R code for analysis and visualization are available in an open online repository. Instances of emergent metabolism were classified by comparing metabolite presence/absence calls between single and double spent media conditions. We describe our method in the Supplementary Materials and Methods. The custom R script used to classify cases of emergent metabolism is also available in the online repository (see Code and data availability).

### Genetic and metabolic similarity analysis

We used the Jaccard distance (1—Jaccard similarity coefficient) to quantify the distance between NOG annotation sets for all pairs of ASF members. We converted the metabolomics profiles (all 85 metabolites) for each species to lists of metabolites which were consumed (*z*-score <2) or produced (*z*-score >2) and calculated the Jaccard distance between all pairs of spent media profiles. The Python script used for this analysis is available in an online repository (see Code and data availability).

### Code and data availability

Detailed methods for scanning electron microscopy, colony imaging, media preparation, and NMR metabolic profiling can be found in the Supplementary Materials. Our data and analysis scripts are available at the following repository: http://mbi2gs.github.io/asf_characterization/.

Some large analysis output files and annotation files for metagenomic data are excluded due to file hosting size limitations, but are available upon request from the authors or can be generated using the indicated raw data, HMMer, associated eggNOG files, and scripts in the repository.

## Results

### Development of a defined medium

We developed a growth medium with defined chemical composition that supports the anaerobic growth of all ASF members (excluding *Mucispirillum* ASF457). This novel, defined medium is based on standard LB medium, supplemented with minerals, salts, and components commonly added to support growth of anaerobes (see detailed formulation in the Supplementary Materials). LB is not generally considered a ‘chemically defined' medium because of complex ingredients such as yeast extract. However, previous research has identified the components of LB to a degree suitable for computational metabolic models and metabolomics purposes (Overbeek *et al.*, 2005; Oberhardt *et al.*, 2008). We confirmed the presence of the majority of expected metabolites using NMR spectroscopy (Supplementary Table 1).

### Morphology and appearance

We describe the cellular and colony morphologies of all eight ASF members in the Supplementary Materials (Supplementary Figures 1 and 2).

### ASF compared with ‘Wild-Type' murine microbiome

**Figure 1:**
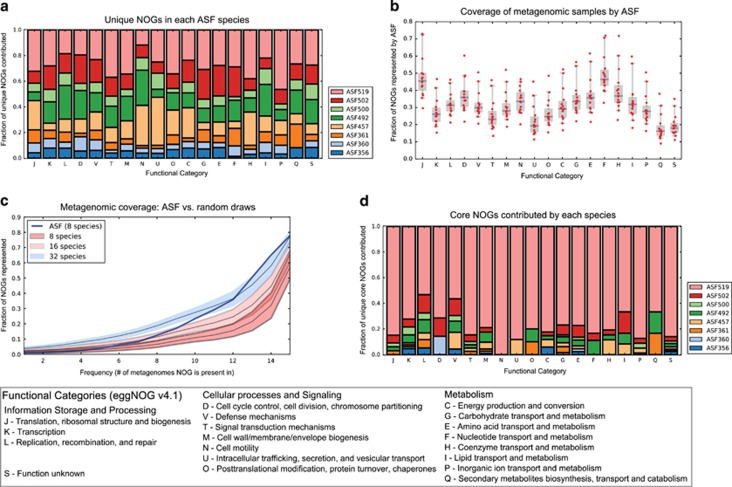
Comparative analysis of the ASF and wild microbiomes. (**a**) The unique contribution of each ASF species to the ASF metagenome is relatively evenly distributed, with the unique contribution of each species being roughly proportional to genome size. Unique NOGs are those present in only 1 ASF species. (**b**) Coverage of the 15 wild mouse fecal metagenomes by the ASF divided by NOG functional category. Coverage indicates how representative the ASF metagenome is of wild mouse metagenomes. Coverage of individual metagenomic samples is represented by red circles, median coverage is shown as a blue line within boxes, boxes extend to mean±1 s.d., and whiskers extend to 5th and 95th percentiles. Across all categories, the ASF overlaps with ~35% metagenomic NOGs. (**c**) Coverage of metagenomic NOGs by the ASF and random microbial consortia. Random consortia mimic the phylum-level distribution of the most abundant species in the mouse gastrointestinal tract. The *x*-axis indicates the number of metagenomes in which the NOGs are present. Coverage of metagenomic NOGs by random consortia of 8, 16 and 32 species (dark to light shading, respectively) are indicated as median lines surrounded by 5th/95th percentile distributions. The ASF covers core metagenomic NOGs (core NOGs occur in all 15 samples) better than any combination of 8 or 16 species and better than the median of 32 species. (**d**) Unique contribution of each ASF species to core metagenomic NOGs. *Parabacteroides* ASF519 contributes the majority of core NOGs in every category.

We annotated the ASF genomes using the eggNOG database and identified unique genetic content within each species (Figure 1a). Non-supervised Orthologous Groups (NOGs) are clusters of highly similar protein sequences, where proteins within each cluster generally share the same function. We found that all eight species possess unique NOGs in proportion to genome size (Wannemuehler *et al.*, 2014). We next compared the composite metagenome of the ASF to the NOGs found in 15 wild murine metagenomes (Figure 1b). We found that the composite ASF metagenome overlaps with the murine microbial metagenome by ~35% in each functional category. Given that the ASF was developed specifically as a surrogate murine microbiome, we hypothesized that the ASF would share key functions with wild-type microbiomes; functions which would be less common in random microbial consortia. We compared the composite ASF metagenome with 10 000 random microbial consortia with similar taxonomic composition (Figure 1c). We sorted NOGs by sample frequency (that is, presence in 1–15 metagenomic samples; frequency distribution shown in Supplementary Figure 3) and determined a core group of NOGs that occurred in all 15 wild murine metagenomic samples (3611 NOGs out of 135 013 unique NOGs observed). Surprisingly, the ASF shares more gene content with the wild metagenomes than any random 8-species consortia. Larger random consortia approach (16 species) and exceed (32 species) the ASF coverage of the wild metagenomes. However, the ASF maintains better coverage of core NOGs (those that occur in all 15 wild microbiomes) than any 16-species consortia and the median of 32-species consortia. In addition, we found that replacing a Bacteroidetes in the random consortia with *Mucispirillum* ASF457 (a member of the phylum Deferribacteres) decreased the coverage of core NOGs (Supplementary Figure 4), demonstrating that the Deferribacteres phylum does not explain the superior coverage by the ASF. The ASF contains 2820 of the 3611 (78.09%) core NOGs. Of these 2820 core NOGs, 1283 (45.50%) are unique to a single ASF species. Of these unique NOGs 1036 (80.75%) are contributed by *Parabacteroides* ASF519, representing the majority of unique core NOGs in every functional category (Figure 1d). These findings suggest that *Parabacteroides* ASF519 is primarily responsible for the ASF's high coverage of the core wild murine metagenome.

### Individual growth characteristics

Each ASF member grew (excluding *Mucispirillum* ASF457) in fresh supplemented LB medium (Supplementary Figure 5). *Lactobacillus* ASF361, *Parabacteroides* ASF519 and *Clostridium* ASF356 grew most rapidly, while *Pseudoflavonifractor* ASF500, *Eubacterium* ASF492 and *Clostridium* ASF502 grew most slowly. Taxonomic relatedness did not necessarily predict growth rates well, given that *Clostridium* ASF356 (a fast grower) and *Clostridium* ASF502 (a slow grower) are both members of the genus *Clostridia*. Similarly, the two *Lactobacilli*, *Lactobacillus* ASF360 and *Lactobacillus* ASF361, vary drastically in growth rate—*Lactobacillus* ASF361 grew more quickly and to a higher density in liquid and on solid media.

### Interactions characterized using spent media

**Figure 2:**
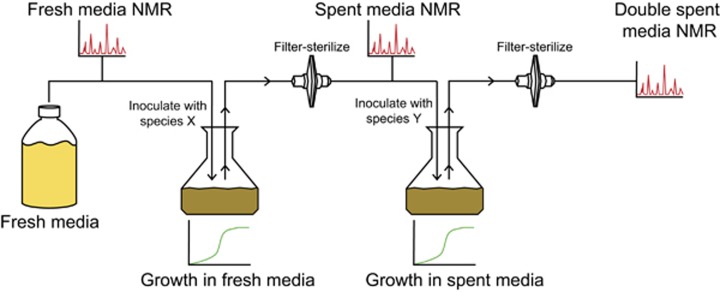
Spent media experimental setup. Each ASF member species was grown independently in fresh growth medium. Growth was monitored for 70 h by optical density, which allowed for comparison of growth rates between species. The supernatant from these first cultures was filter sterilized to produce ‘spent media', which was subsequently profiled by NMR spectroscopy and compared to the fresh growth medium. This initial metabolomics analysis identified the metabolites utilized and by-products produced by each species. For the second round, each ASF species was inoculated into spent medium from the other species. Growth was monitored and compared to growth in fresh medium. The supernatant from this second round (‘double spent media') was filter sterilized and compared to the spent medium from which it originated to identify further metabolites that were used or consumed.

We characterized directional, species-species interactions by screening all pairs of ASF members through a series of spent media experiments (Figure 2). In brief, spent medium was prepared for each species by growing it in fresh liquid media for 70 h. We use the notation ‘spentXXX' to indicate the supernatant resulting from growth of ASFXXX (for example, ‘spent356' to indicate the spent media resulting from growth of *Clostridium* ASF356). Having reached stationary phase, the supernatant from the culture was filter sterilized. This resulting spent medium was used to culture each ASF member in turn. The loss of some substrates and addition of new by-products from the first species influenced the growth of subsequent species. The supernatant resulting from the growth of a second species in the spent media from a previous species is referred to as ‘double spent media'.

#### Growth inhibition

No species was able to grow in its own spent medium, which is consistent with the expectation that a species has exhausted a media environment once it has entered stationary phase (Supplementary Figure 5). The majority of interactions resulted in decreased growth or completely stifled growth in the second species. *Parabacteroides* ASF519 was able to grow in the spent media from most other member species with the exception of spent medium from *Lactobacillus* ASF361. *Lactobacillus* ASF360 and *Pseudoflavonifractor* ASF500 were unable to grow in the spent media from any other species. Marginal growth was observed with *Lactobacillus* ASF361 grown on spent492 (that is, spent media produced by *Eubacterium* ASF492). *Lactobacillus* ASF361 prevented growth of all other members, while spent media from *Clostridium* ASF356 and *Parabacteroides* ASF519 prevented growth of all species with the exception of each other.

#### Metabolic profiling of spent media

NMR spectra were obtained for all fresh, spent, and double spent media conditions (Supplementary Figure 6 and Supplementary Metabolomics Plots). Across all samples, 85 NMR peaks exhibited significant variation (Supplementary Figure 6). We were able to confidently map 36 peaks to known metabolites in our library of reference spectra.

**Figure 3:**
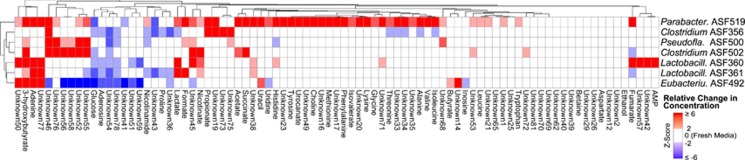
Relative changes for 85 NMR peaks in single spent media samples. NMR peak integrals are proportional to metabolite concentrations. Relative changes in peak integrals are displayed as *z*-scores relative to fresh media, with zero (white) indicating that the metabolite concentration is the same as in fresh media. Values >2 standard deviations above (red) or below (blue) zero indicate concentrations higher or lower than fresh media, respectively. *Z*-scores ⩽−6 or ⩾6 are displayed as −6 or 6, respectively. Rows correspond to individual ASF members. For example, the first row indicates the metabolite *z*-scores relative to fresh media after the growth of *Parabacteroides* ASF519.

Significant metabolic differences were observed among the ASF members growing in fresh media (Figure 3). *Parabacteroides* ASF519 consumed the fewest metabolites (only glucose, Unknown 41 and Unknown 43), while it produced many other metabolites including amino acids (alanine, glycine, histidine, isoleucine, leucine, lysine, methionine, phenylalanine, threonine, tryptophan, tyrosine and valine). *Clostridium* ASF356 uniquely consumed isoleucine, valine, alanine, threonine and lactate and some unidentified metabolites (Unknowns 33, 34, and 35). *Eubacterium* ASF492 was the only species to consume uridine and several unidentified metabolites (Unknowns 45, 46, 56, and 58), while *Pseudoflavonifractor* ASF500 was the only consumer of histidine. *Eubacterium* ASF492 was the only species to produce butyrate in fresh media. Glucose was clearly consumed by all species except *Pseudoflavonifractor* ASF500. Nicotinamide, adenosine and two unidentified metabolites (Unknowns 54, 78) were also consumed by most species. While all spent media was acidic, *Lactobacillus* ASF361 produced the most acidic spent media (Supplementary Table 2). The reproducibility of these NMR-based observations was confirmed by comparison with an independent set of biological replicates and subsequent NMR metabolomic profiling (Supplementary Metabolomics Plots).

**Figure 4:**
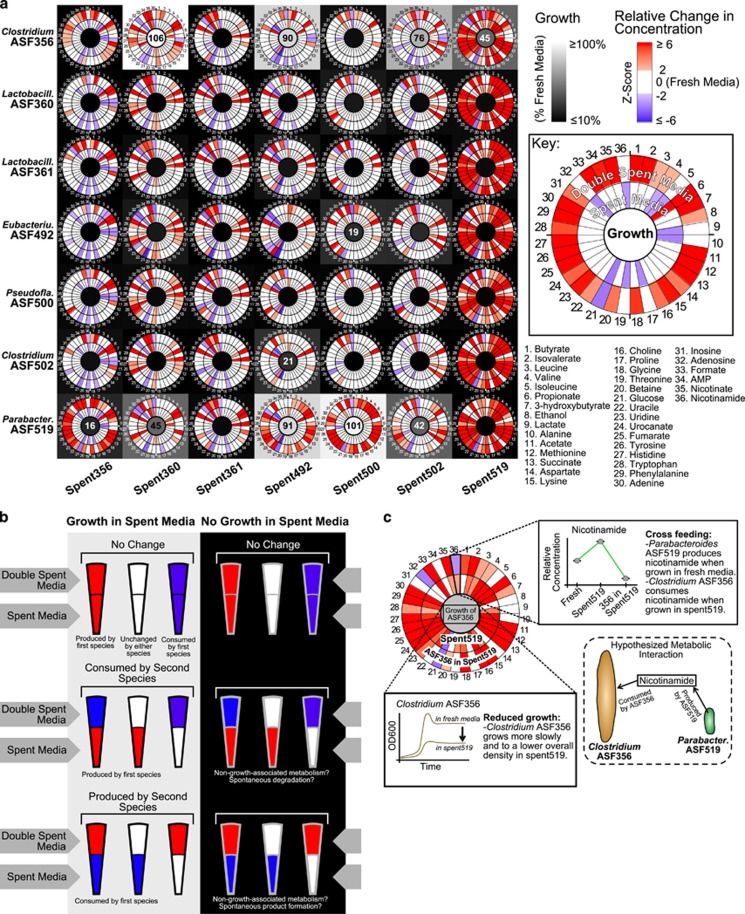
Metabolomics analysis of all media conditions. (**a**) The black and white heat map underlying the figure indicates the growth achieved under spent media conditions compared to fresh media conditions. Growth is quantified using the area under the curve (AUC), indicated as a percentage of the AUC when a species is grown in fresh media. White indicates growth equal in rate and density to fresh media conditions (for example, *Parabacteroides* ASF519 grown in spent500), while black indicates complete inhibition of growth (for example, *Parabacteroides* ASF519 grown in spent361). For species that achieved an AUC of at least 10%, we annotate the AUC in the center of the appropriate tile. Circular heat maps within each cell display the metabolomics profiles for the spent media in that column (inner ring) and the ‘double spent' media (the result of growing the species from that row in the spent media from that column; outer ring). Metabolite concentrations are quantified as z-scores relative to fresh media, and are displayed as circular heat maps. Zero (white) indicates no significant change from fresh media. Values >2 standard deviations above (red) or below (blue) zero indicate concentrations higher or lower than fresh media, respectively. For example, *Eubacterium* ASF492 grown in spent500 is able to grow (although not optimally—only 19% AUC). *Eubacterium* ASF492 produces a butyrate when grown in spent500, which can be seen by comparing the inner circle (relative concentration of butyrate in spent500) to the outer circle (higher relative concentration of butyrate in media after growth of *Eubacterium* ASF492). All the metabolomics data are available in greater detail in the Supplemental Metabolomics Plots. (**b**) Qualitatively, there are 18 possible scenarios when comparing double spent media to the spent media from which it was derived. In general, a metabolite can increase, decrease, or remain the same, and the interpretation of that behavior is related to whether there was observed growth in that condition. For example, a metabolite that is depleted by the first species and remains so (no change) under a no growth condition may indicate a metabolite which was required for growth of the second species. Alternatively, if a metabolite is produced by the first species, consumed by the second and growth is observed, this constitutes evidence for cross-feeding. (**c**) An example: Nicotinamide is elevated in spent519 (inner ring) and depleted when *Clostridium* ASF356 is grown in spent519 (outer ring). *Clostridium* ASF356 does grow (AUC=45%), so we hypothesize that in a co-culture, *Clostridium* ASF356 would benefit from *Parabacteroides* ASF519 producing nicotinamide.

We interpreted the double spent samples by comparing them with the spent media from which they were derived (Figures 4a and b). If a species was able to grow in a spent media, the associated changes in metabolite abundances can be attributed to metabolic activity of that species. Of interest are those metabolites which may contribute to cross-feeding or competition in a co-culture setting. If growth is inhibited in a spent media, the metabolite profiles of the spent media can indicate compounds required for growth, or alternatively, toxic compounds.

Of the 3570 metabolite comparisons between spent and double spent media conditions, 2695 (75%) were unchanged between the spent and double spent conditions (Table 1). Of these, 2081 (77%) unchanged metabolites were associated with conditions where the second species did not grow. Of the 2550 comparisons where the second species did not grow, 469 (18%) changed. If a metabolite changed between spent and double spent conditions, usually it increased (717 instances of 875 changed, or 82%).

Cases of potential cross-feeding were rare, where the second species grew and simultaneously consumed a metabolite produced by the first species (32 instances of 875 changed, or 4%). *Clostridium* ASF356 was able to grow in spent519 (area under a growth curve=45% of fresh media growth; Figure 4c) and was the condition most enriched for cases of potential cross-feeding (10 of 85 possible metabolites, or 12%). As an example of potential cross-feeding, *Parabacteroides* ASF519 produced nicotinamide when grown in fresh media, and *Clostridium* ASF356 appears to have consumed the nicotinamide in the spent519 media (Figures 4a and c). Given these data, we hypothesize that cross-feeding may occur in a co-culture setting such that *Clostridium* ASF356 would consume nicotinamide produced by *Parabacteroides* ASF519. Between spent519 and *Clostridium* ASF356 grown in spent519, similar cross-feeding-like profiles are observed for alanine, isoleucine, lactate, threonine, uracil and several unidentified metabolites (Unknowns 35, 52, 55, and 76).

There were 856 instances of emergent metabolism (of 3570 possible), 168 of which occurred in only one condition. For instance, we observed cases of emergent biosynthesis, such that a species produced a given metabolite only when grown in the spent media of another species. *Parabacteroides* ASF519 produced butyrate, betaine, and several unidentified metabolites (Unknowns 2, 12, 14, 36, 39 and 40), but only when grown on spent356 or spent500 (Supplementary Figure 6). There were several cases where the emergent phenotype occurred in a single condition. For example, *Clostridium* ASF356 only produced aspartate, lysine, methionine, phenylalanine, succinate, tyrosine and several unidentified metabolites (Unknowns 23, 25, 26 and 65) when grown in spent360. Alternatively, while *Parabacteroides* ASF519 produced 3-hydroxybutyrate in every other media condition, when grown in spent502 it switched to consuming 3-hydroxybutyrate. The complete list of emergent, metabolic observations is available in Supplementary Data 2.

##### Genetic distance associated with variance in metabolic distance

**Figure 5:**
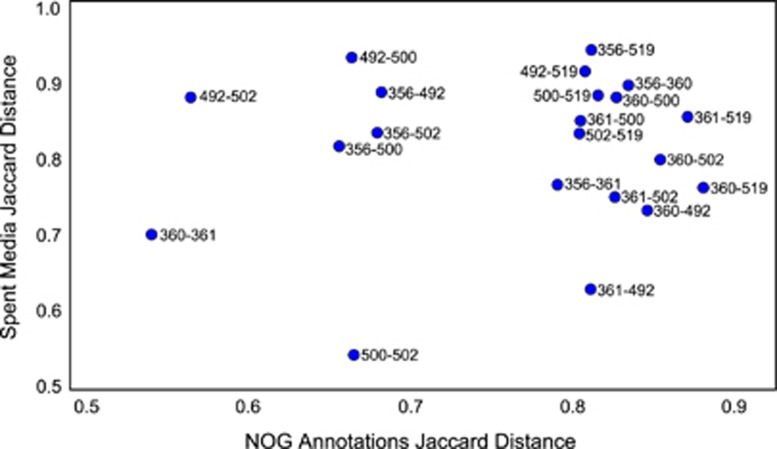
Genetic distance associated with greater variance in metabolic distance. We quantified the genetic distance between all species pairs using the Jaccard distance between the NOG annotation sets. We similarly quantified the distance between the spent media metabolomics profiles for all pairs of ASF members. Genetic similarity is not strongly correlated with metabolic state under these conditions. Points are labeled with the ASF identifiers for the species pair.

We quantified the genetic distance between all pairs of ASF members using the Jaccard distance between the NOG annotations for each pair. Using the same metric, we quantified the distance between spent media metabolomics profiles for all pairs of ASF members. We found that genetic similarity is not strongly correlated with metabolic state under these conditions (Figure 5). Indeed, some pairs of closely related species (for example, *Lactobacillus* ASF360 and *Lactobacillus* ASF361) were more different in terms of spent media profiles than some more distant pairs (for example, *Pseudoflavonifractor* ASF500 and *Clostridium* ASF502). Furthermore, we performed a correlation analysis and identified 11 079 NOG-metabolite pairs which were statistically significant (Spearman's correlation and Bonferonni multiple testing correction with *n*=160 746; Supplementary Figure 7). After excluding unique NOGs and metabolites which were consumed or produced by a single species, 458 correlations were significant.

## Discussion

We present a novel approach to characterizing microbial communities *in vitro*, and the results of applying this approach to gain insights into a model microbial community known as the ASF. Through a bioinformatics analysis, we found that the ASF is far more representative of wild microbiome functionality than random consortia of similar or larger size. Through a spent media screen, we found that cross-feeding interactions are relatively rare, while non-growth-associated and emergent metabolism are relatively common. These, together with the rest of our findings, form the beginnings of a rich knowledge base which increases the utility of the ASF as a model gut community.

A primary outcome of this work is standardization of ASF resources. First, our morphological descriptions accompanied by images, all gathered under the same conditions, provide a reference for future researchers. Comparing morphology to references such as these will improve reproducibility and support the discovery of new phenotypes. Second, we developed a chemically defined LB-based medium which simplifies metabolic profiling, and ongoing efforts to build genome-scale metabolic network reconstructions for the ASF members. One drawback of the new LB-based medium is that it does not support the growth of *Mucispirillum* ASF457 (*M. schaedleri*), which is difficult to grow effectively, even in complex media (Sarma-Rupavtarm *et al.*, 2004). We attempted, unsuccessfully, to identify media components which would allow *Mucispirillum* ASF457 to grow, including the addition of porcine mucin, given the fact that *Mucispirillum* ASF457 colonizes the mucous layer in the murine colon (Robertson *et al.*, 2005). We also attempted to grow *Mucispirillum* ASF457 in the spent media of other ASF members. This is an area for future research (Supplementary Data 3 includes a list of gene annotations missing from *Mucispirillum* ASF457 that are present in all other ASF members). Despite this shortcoming, the new media successfully enabled metabolic profiling of the remaining seven ASF members and their interactions.

The presented genomic analysis of the ASF vastly expands our knowledge of how the ASF relates—on a functional level—to more complex microbiomes. Indeed, the ASF is far simpler than a wild-type microbiome in terms of both species and genetic composition. We found that *Parabacteroides* ASF519 is a major contributor to the unique qualities of the ASF, with impressive coverage of genes and metabolic activities that may be vital in the wild mouse microbiome. Additional studies are needed, which reach beyond coverage of functional orthologs, to better understand both the essential and redundant roles played by each ASF species.

Traditional co-culture experiments have several drawbacks which make it challenging to determine the mechanism underlying interactions between two species. These include difficulties determining which species utilized or produced a given metabolite (Ghosh *et al.*, 2014), and measuring growth of individual species which requires tools with lower temporal resolution and much higher costs than optical density (Arquiza and Hunter, 2014; Novakova *et al.*, 2014; Wolfe *et al.*, 2014; Aghababaie *et al.*, 2015). Computationally inferring interactions from metagenomic data generally cannot resolve interaction directionality, but rather is limited to identifying correlations between species abundances (Faust and Raes, 2012). Spent media experiments resolve some issues confronted in co-culture experiments and computational inference. By separating interactions into two steps (Figure 2), it is simple to infer interaction directionality, and straightforward to generate hypotheses about underlying mechanisms (Lawrence *et al.*, 2012; Khare and Tavazoie, 2015). Growth measurements can be gathered at high resolution with metrics such as optical density because only a single species is growing. It should be noted, that spent media experiments do not allow for cell-to-cell contact or dynamic signaling between species, which may otherwise be relevant in co-culture or *in vivo* (Sibley *et al.*, 2008). Moreover, the nature of interactions can be context-specific, such that interactions identified in this spent media screen are not definitive for all conditions (Klitgord and Segrè, 2010). Finally, while we utilized 100% spent media in this study, we expect that a wider range of growth phenotypes will be observable by using spent media dilutions or by creating ‘partially spent media' such that there are sufficient nutrients to support growth, but molecules from the first species would still be able to influence the second species. This is a promising direction for future research.

Our interpretation of these spent media experiments relies first on comparing the growth dynamics of a given species in both fresh media and spent media (Supplementary Figure 5). Subsequent metabolic profiling (Figure 4) identified specific compounds hypothesized to have a role in causing the observed interaction dynamics. In a similar spent media experiment between environmental isolates of leaf-degrading bacteria, it was found that natural isolates engage in less cross-feeding than isolates evolved together for several generations *in vitro* (Lawrence *et al.*, 2012). While the ASF has evolved for many generations as a single community within mice, the defined media environment used in our *in vitro* experiments is very different from the murine gut environment, and so it is not surprising that we observed few instances of cross-feeding. An interesting future direction would be to evolve the ASF *in vitro* in the defined medium, after which we would predict that more cross-feeding would be observable. Our observation that emergent metabolic phenotypes are common between ASF members (at least one emergent phenotype identified between all pairwise species interactions) agrees with recent computational and *in vitro* work demonstrating that the vast majority of microbial pairs and media conditions exhibit emergent biosynthetic behaviors (Chiu *et al.,* 2014; Traxler *et al.*, 2013). Also of interest, we observed 469 instances in which metabolite relative abundances changed despite an absence of growth (for example, *Lactobacillus* ASF361 grown in spent500). One explanation is that in these cases, inoculated cells are metabolically active without active cell division (Gefen *et al.*, 2014). It is notable that most species grew slower (or not at all) and to a lower overall density in spent media (Figure 4 and Supplementary Figure 8). It is unlikely that another species could produce a mixture of compounds more growth-promoting than already found in rich media.

We found that the similarity of genetic annotations between any pair of ASF members was not strongly correlated with the metabolic phenotypes of those same pairs (Figure 5). This result is not surprising given that the relationship between the phylogenetic distance and the metabolic capabilities of two species can be modeled by an exponential, the relationship is neither linear nor strong (Plata *et al.*, 2014). Furthermore, we identified several cases where the NOG presence/absence distribution was significantly correlated with the consumption or production of specific metabolites (Supplementary Figure 7). However, because there are so few species, the vast majority of these significant correlations are between NOGs found in a single species and the metabolites which were uniquely consumed or produced by that species. These results reinforce the need for more sophisticated approaches to linking genotype to phenotype, for example, using comparative metabolic network modeling (Bartell *et al.,* 2014).

**Figure 6:**
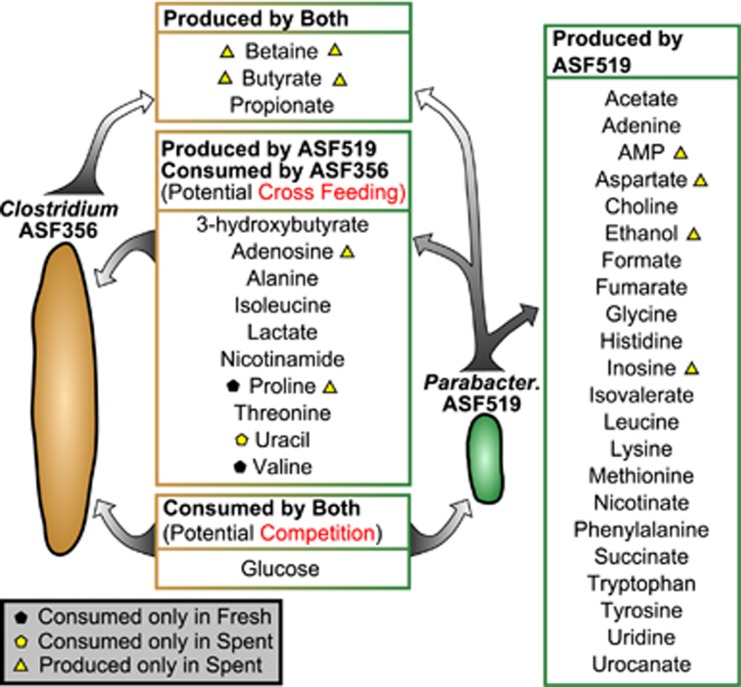
Inferred metabolic interactions between *Clostridium* ASF356 and *Parabacteroides* ASF519. *Clostridium* ASF356 and *Parabacteroides* ASF519 were able to grow in many more spent media conditions than other species, including spent media from each other. We combined evidence from four media conditions including spent356, spent519, *Clostridium* ASF356 grown in spent519 and *Parabacteroides* ASF519 grown in spent356 to form our hypothesis of the metabolic interactions that would occur in co-culture. Black pentagons indicate metabolites that are consumed only from fresh media, yellow pentagons indicate metabolites that are consumed only from spent media, and yellow triangles indicate metabolites that are produced only in spent media. Shapes on the left correspond to Clostridium ASF356 while those on the right correspond to Parabacteroides ASF519. In general, *Parabacteroides* ASF519 produces many more compounds than *Clostridium* ASF356, while *Clostridium* ASF356 consumes many more compounds than *Parabacteroides* ASF519. Both species consume—and would be expected to compete for—glucose. Both species produce propionate in abundance, while both species also produce butyrate and betaine, but only when grown in the spent media from the other. For clarity, we have excluded unidentified NMR peaks from this figure.

Of the seven ASF members that grew in the supplemented LB medium, *Clostridium* ASF356 and *Parabacteroides* ASF519 grew most rapidly and to the highest overall density (Supplementary Figure 5). Furthermore, both species grew in spent media from each other (Figure 4). A summary of the data for *Clostridium* ASF356 and *Parabacteroides* ASF519 highlights competition for glucose and many opportunities for cross-feeding (Figure 6). *Clostridium* ASF356 consumed a far more diverse assortment of metabolites, while *Parabacteroides* ASF519 consumed very few. *Parabacteroides* ASF519 has a more diverse metabolic output than *Clostridium* ASF356. Considering ASF spatial distribution *in vivo*, *Clostridium* ASF356 is more abundant in the cecum and *Parabacteroides* ASF519 is the most abundant ASF member in the colon (Sarma-Rupavtarm *et al.*, 2004). *Parabacteroides* ASF519 appears to be a scavenger, growing robustly in the distal colon where the ability to produce essential biomass components from few inputs is an advantage.

The observation that *Parabacteroides* ASF519 required few substrates is partially explained by its large genome size (6.87 Mb), the largest of the ASF. We would expect large genome size to correlate with greater biosynthetic capacity, knowing that smaller genomes correlate with auxotrophy (D'Souza *et al.*, 2014). The metabolic characteristics of *Parabacteroides* ASF519 are also interesting in light of our comparison of random consortia to the ASF: the core functions found in fecal metagenomes were covered best by *Parabacteroides* ASF519 (Figure 1d), which allowed the ASF to out-perform much larger microbial consortia (Figure 1c). Naturally, the large genome of *Parabacteroides* ASF519 could lead to more coverage of the core metagenome. However, the next two largest genomes from *Eubacterium* ASF492 (6.51 Mb) and *Clostridium* ASF502 (6.48 Mb) do not come close to the same coverage, nor do these species display the same prolific biosynthetic activity under these *in vitro* conditions. *Parabacteroides* ASF519 is an unexpectedly vital contributor to the ASF metagenome and metabolic activity.

Our analysis of the ASF metagenome and spent media experiments have produced a profile of ASF genetics and metabolism which will enable future research to leverage knowledge of the unique qualities of the ASF. As an example of how the ASF can be used advantageously, a recent study leveraged an understanding of ASF metabolism to engineer a microbiome which improved survival of mice with hepatic injury (Shen *et al.*, 2015). An example where more detailed information about ASF metabolism would have been highly relevant, a recent study colonized gnotobiotic mice with a subset of the ASF in an attempt to prevent butyrate production (Donohoe *et al.*, 2014), knowing that the full ASF community does produce butyrate *in vivo* (Smith *et al.*, 2013). That subset correctly excluded *Eubacterium* ASF492, but included *Parabacteroides* ASF519, which we found to also produce butyrate. Future experiments excluding *Parabacteroides* ASF519 from ASF-colonized mice will enhance our understanding of its impact on mouse health and metabolism, and could shed light on the role of similar species in the gastrointestinal tract of humans. The metabolomics profiles presented in this study indicate nutritional supplements which could be used as pre-biotics in ASF-colonized mice. Indeed, greater understanding of the ASF increases its utility as a testing ground for validating strategies for the development of microbiome-targeted therapies.

The ASF is a unique microbial community with a long history of use in murine models, with untapped potential to become a highly characterized model microbiome. Our characterization of ASF morphology, functional genetic content, growth, metabolism and interactions lays a strong foundation for future research into gut ecology and efforts to engineer the gut microbiome to improve health.

## Figures and Tables

**Table 1:** Classification of metabolite profiles between spent media and double spent media (known metabolites only)

	*Growth*			*Subtotal*	*No Growth*			*Subtotal*	*Totals*
No Change	High	Medium	Low	614	High	Medium	Low	2081	2695
	146	416	52		460	1426	195		
Lower in double spent	High to Low	High to Medium	Medium to Low	73	High to Low	High to Medium	Medium to Low	85	158
	2	30	41		3	43	39		
Higher in double spent	Low to High	Low to Medium	Medium to High	333	Low to High	Low to Medium	Medium to High	384	717
	9	51	273		10	85	289		
Totals	157	497	366	1020	473	1554	523	2550	3570

Categories correspond to those presented in Figure 4b. Metabolite relative abundance indications: ‘High' indicates a relative abundance 2 standard deviations above that in fresh media; ‘Med.' indicates an abundance within ±2 s.d. of that in fresh media; ‘Low' indicates a relative abundance 2 s.d. below that in fresh media. For example, where a second species grew in the spent media of a first species (‘Growth' column), there were 9 cases where a metabolite which was ‘low' in the spent media increased to ‘high' in the double spent media (‘Low to High').
