# Supplementary material for: Systems-level metabolism of the altered Schaedler flora, a complete gut microbiota
Source: ISME J. 2016 Nov 8;11(2):426–38. doi: 10.1038/ismej.2016.130 (PMC5270571; doi:10.1038/ismej.2016.130)
Supplement: Supplementary Information [file ismej2016130x1.pdf]

Supplemental Materials: Systems-level metabolism of the Altered Schaedler Flora, a complete gut microbiota

Matthew B. Biggs[1], Gregory L. Medlock[1], Thomas J. Moutinho Jr.[1], Hannah J. Lees[2], Jonathan R. Swann[2], Glynis L. Kolling[3*], Jason A. Papin[1*]

1. Department of Biomedical Engineering, University of Virginia, Charlottesville, VA USA
2. Division of Computational and Systems Medicine, Imperial College, London, UK
3. Department of Medicine, Infectious Diseases, University of Virginia, Charlottesville, VA USA

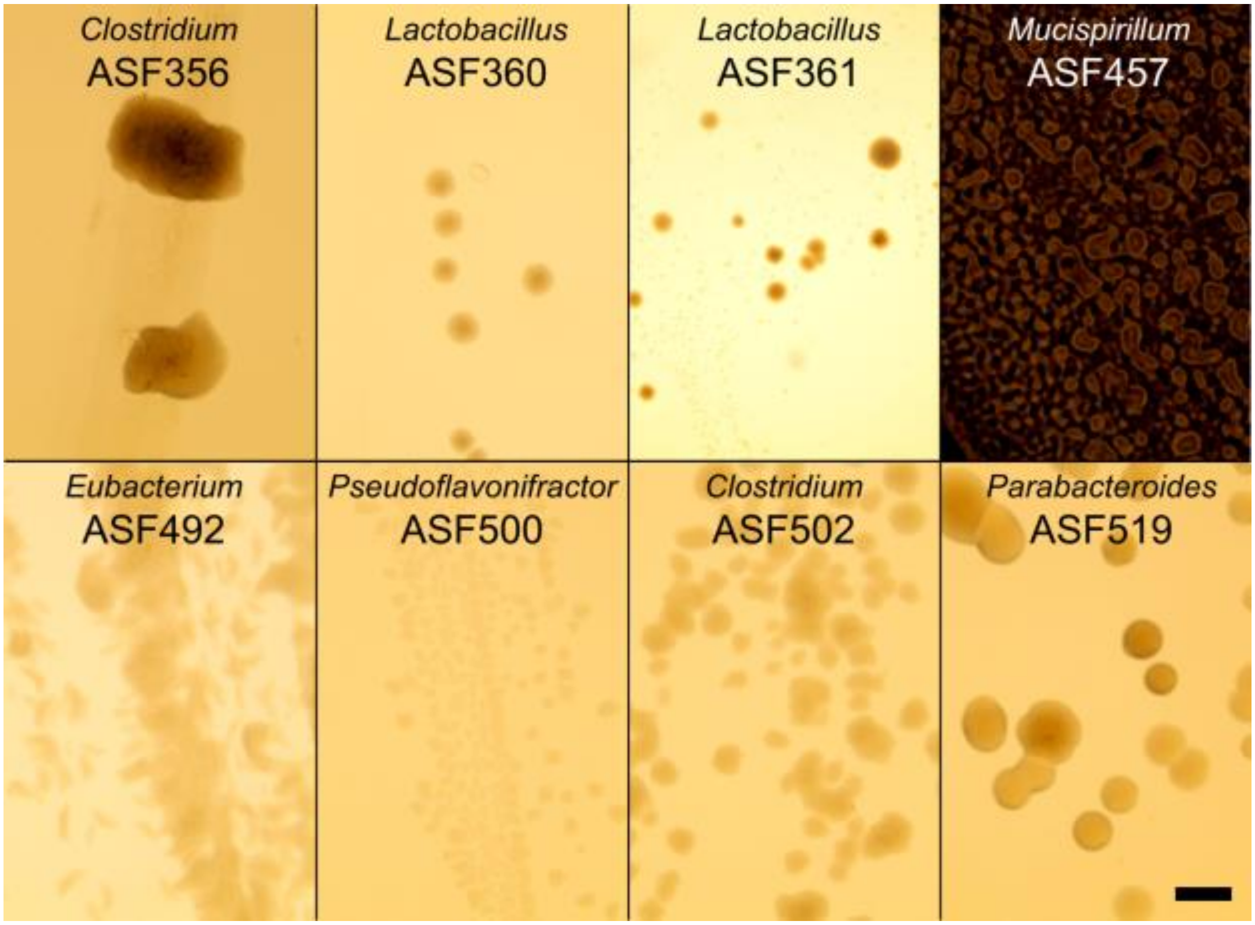

Supplemental Figure 1. Colony morphology of the Altered Schaedler Flora (ASF). Grown on supplemented LB, *Clostridium* ASF356 forms irregularly-shaped, smooth colonies with evident yellow pigmentation in larger colonies at the lobate margins. *Lactobacillus* ASF360 forms small, smooth circular colonies without pigmentation. *Lactobacillus* ASF361 forms moderate to large circular colonies with a rough texture and translucent margins. *Eubacterium* ASF492 forms irregularly-shaped small, smooth colonies with undulate margins without pigmentation. *Pseudoflavonifractor* ASF500 forms numerous circular punctiform colonies without pigmentation. *Clostridium* ASF502 forms numerous circular punctiform colonies without pigmentation. *Parabacteroides* ASF519 forms large circular, glistening

colonies with a convex shape. A slight yellow pigmentation was observed in a few *Parabacteroides* ASF519 colonies. *Mucispirillum* ASF457 grown on supplemented BHI forms a translucent, uneven film. Here, *Mucispirillum* ASF457 was imaged using phase contrast to highlight colony edges. Scale bar represents 1 mm.

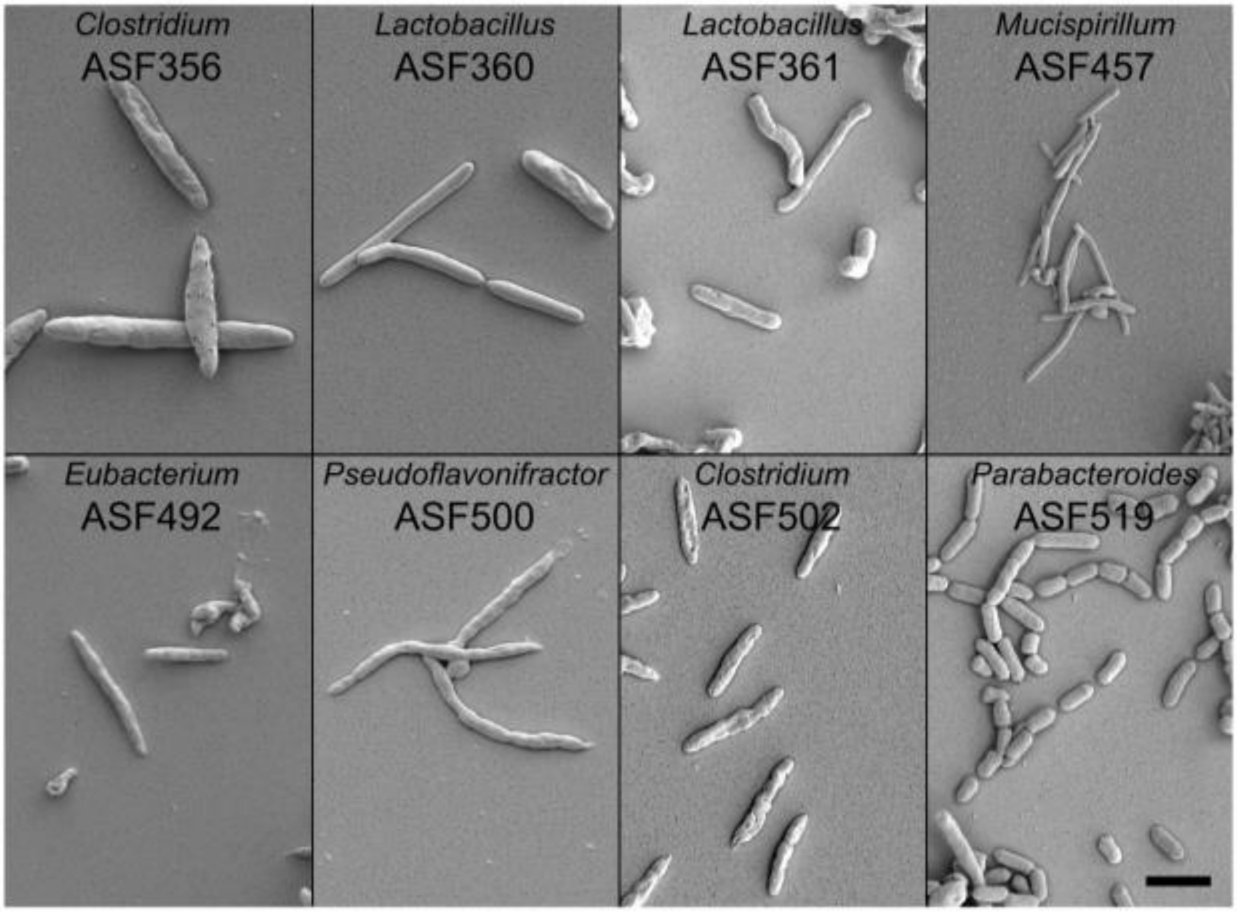

Supplemental Figure 2. Cellular morphology of members of the Altered Schaedler Flora (ASF) on supplemented BHI agar By SEM, *Clostridium* ASF356, *Eubacterium* ASF492, *Pseudoflavonifractor* ASF500, and *Clostridium* ASF502 appear as fusiform rods. *Lactobacillus* ASF360, *Mucispirillum* ASF457 and *Parabacteroides* ASF519 appear as rods, while *Lactobacillus* ASF361 appears as an irregular rod. *Parabacteroides* ASF519 forms chain structures, while other species are generally observed singly or in pairs. *Clostridium* ASF356 is the largest species, on average, and *Parabacteroides* ASF519 is the smallest. Scale bar represents 2 µm.

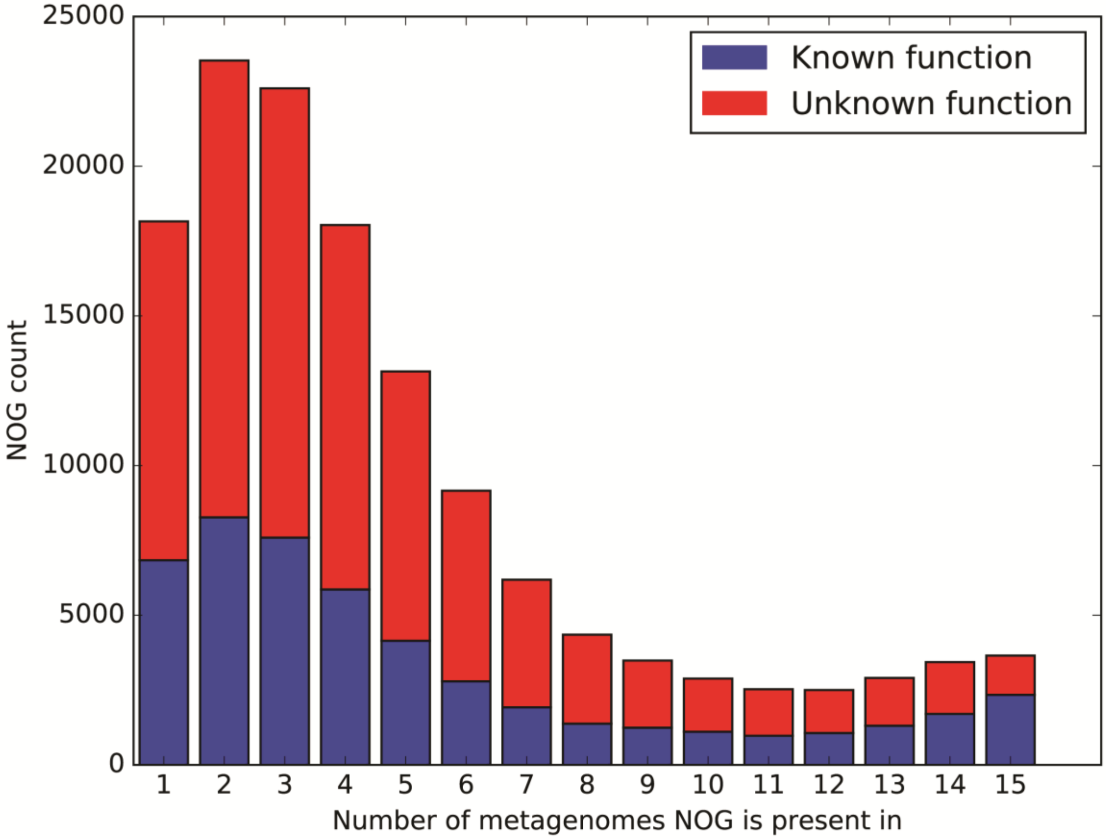

Supplemental Figure 3: Histogram of NOG distribution among 15 metagenomic samples. 135 013 unique NOGs were annotated in 15 metagenomic samples. X-axis indicates the number of samples in which NOGs are present. The distribution is bimodal such that most NOGs are relatively rare (primary peak indicates that many NOGs are found in 6 or fewer samples), while there is a conserved group of "core" NOGs (i.e. NOGs which are present in all samples).

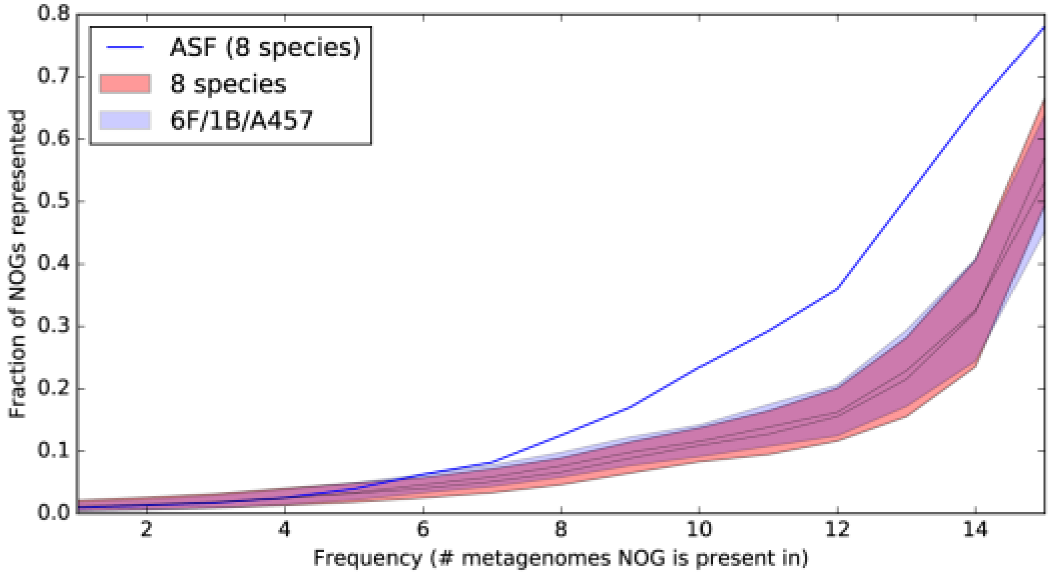

Supplemental Figure 4: Coverage of metagenomic NOGs by the ASF and random microbial consortia which include/exclude *Mucispirillum* ASF457. Random consortia mimic the phylum-level distribution of the ASF. The x-axis indicates the number of metagenomes in which the NOGs are present. Coverage of metagenomic NOGs by random consortia of 8 species is shown as median lines surrounded by 5th/95th percentile distributions. Coverage by consortia composed of 6 Firmicutes and 2 Bacteroidetes is indicated by the light red shaded region. Coverage by consortia composed of 6 Firmicutes, 1 Bacteroidetes and 1 Deferribacteres (*Mucispirillum* ASF457) is indicated by the blue shaded region. Inclusion of *Mucispirillum* ASF457 in the random consortia reduces coverage of core NOGs, indicating that the presence of *Mucispirillum* ASF457 does not explain the ASF's unusually high coverage relative to random consortia.

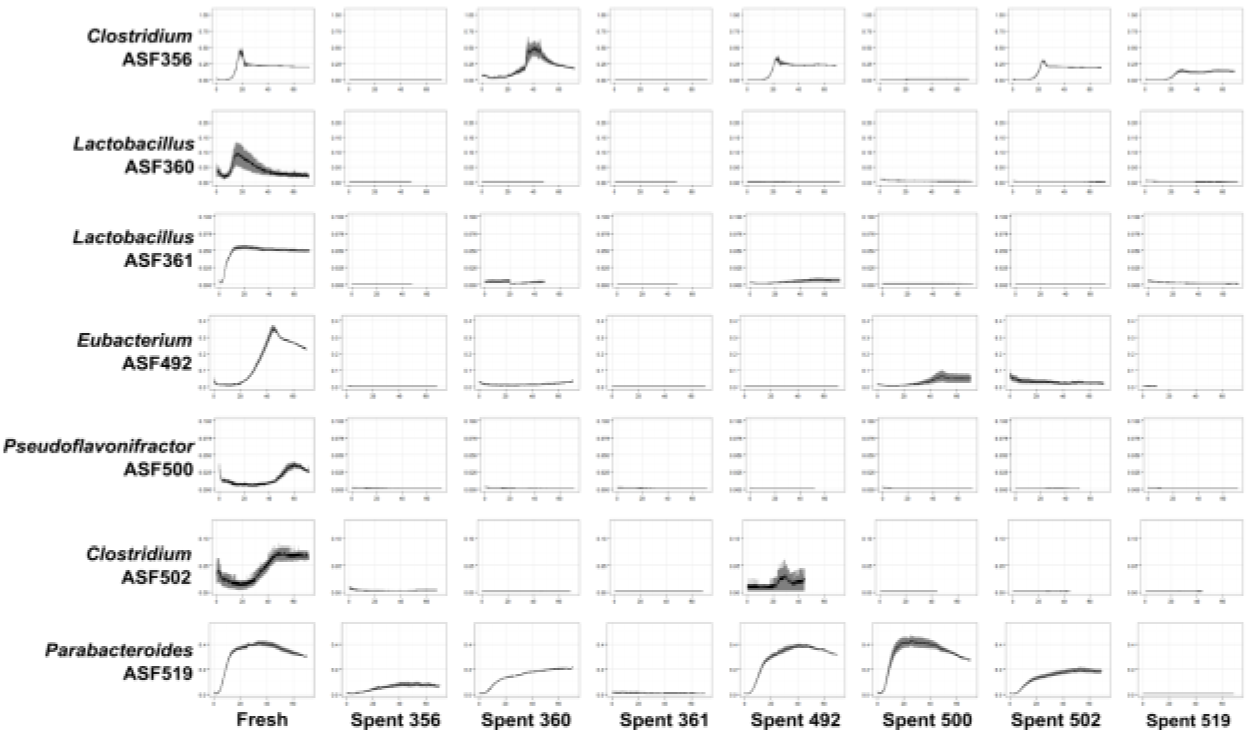

Supplemental Figure 5. Growth curves for all combinations of media and species. Optical density time courses were collected over ca. 70 h at 37°C for all media conditions. The y-axes for all subplots indicate optical density (OD600), while the x-axes indicate time (70 h). Rows indicate the species which is actively growing, and the y-axis limits are consistent within a row. Columns indicate the growth media conditions, where, for example, “Spent356” indicates the spent media obtained after growth of *Clostridium* ASF356. The mean optical density from four replicates is indicated as a black line, while the standard error of the mean is indicated in gray.

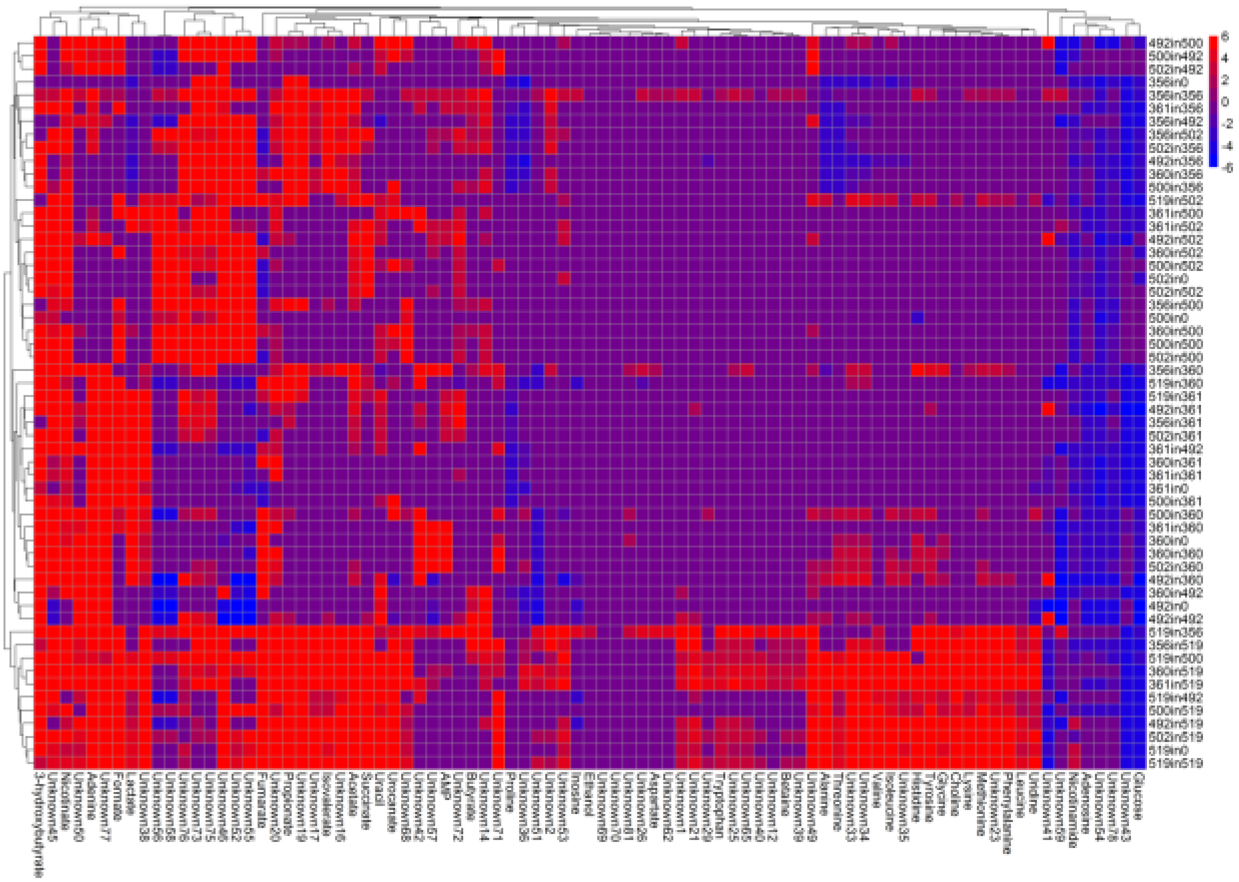

Supplemental Figure 6: Relative changes for 85 NMR peaks in all samples. NMR peak integrals are proportional to metabolite concentrations. Relative changes in peak integrals are displayed as z-scores relative to fresh media, with zero (purple) indicating that the metabolite concentration is that same as in fresh media, >2 standard deviations (red) indicates higher concentration than fresh media, and <2 standard deviations (blue) indicates lower concentration than fresh. Z-scores ≤ -6 or ≥ 6 are displayed as -6 or 6, respectively. The rows are labeled as "GrowthSpeciesID-in-MediaID". For example, the first row indicates the metabolite z-scores relative to fresh media after *Eubacterium* ASF492 grew in spent media from *Pseudoflavonifractor* ASF500. A media ID of "0" indicates fresh media.

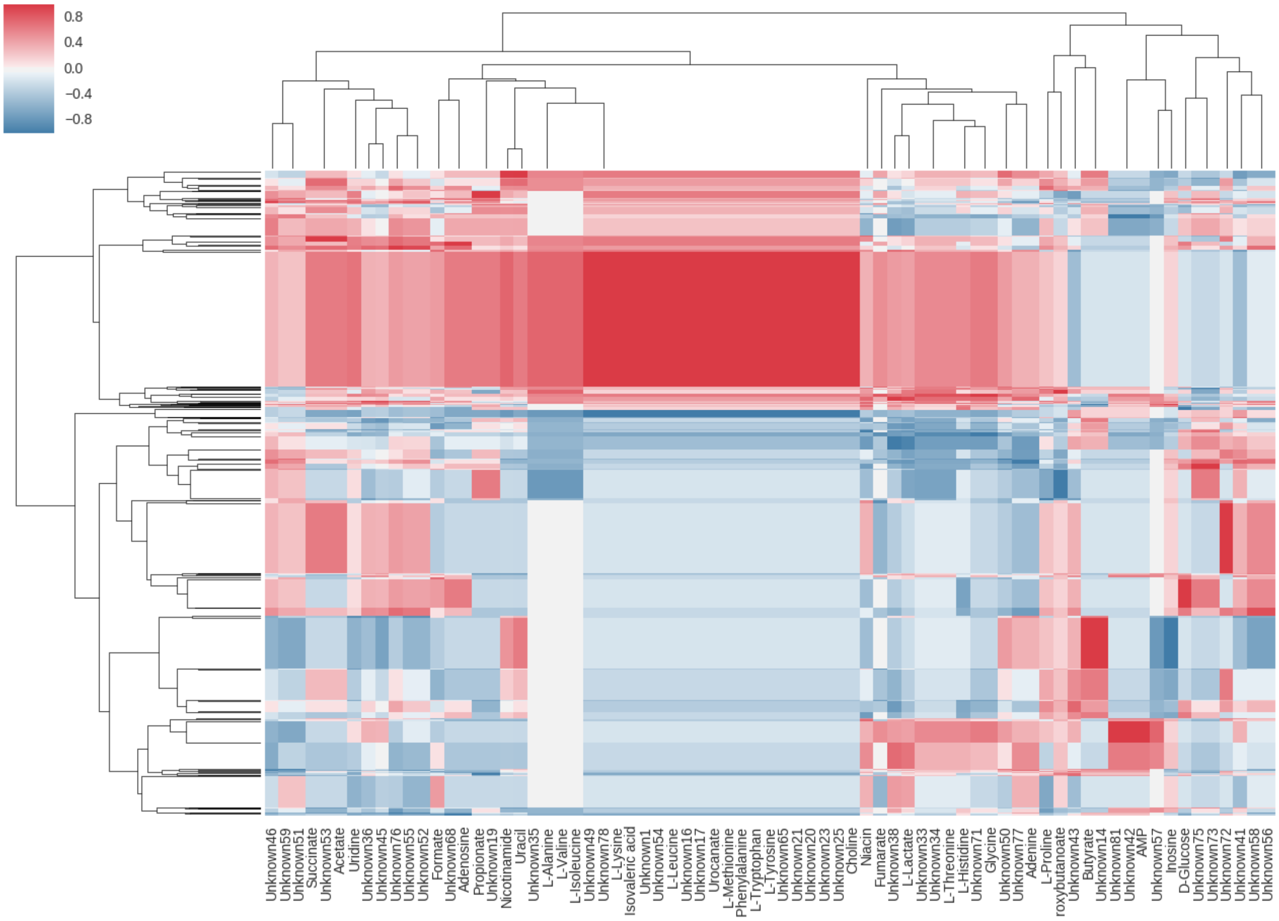

Supplemental Figure 7: Correlation of NOG distribution and metabolite consumption/production patterns in fresh media. The metabolites are represented in the columns, and the NOGs are represented in the rows (NOG identifiers are omitted due to space constraints). This heat map displays the Spearman correlation between NOG presence and metabolite production/consumption (see Supplemental Materials and Methods). Consumption and production were defined as ±2 standard deviations below or above the relative abundance in fresh media, respectively. The Spearman correlation was calculated between all pairs of 2 202 metabolic NOGs in the ASF with each of 73 metabolites. After Bonferroni correction, 11 079 of the 160 746 correlations (6.9%) were statistically significant (p-value $< 3.1 \times 10^{-7}$). However, removing unique NOGs and metabolites which were uniquely consumed or produced by a single species reduced the significant correlations to 458 (0.2%). Red shading indicates a positive correlation (presence of a NOG coincides with production of a metabolite) while blue shading indicates a negative correlation (presence of a NOG coincides with consumption of a metabolite).

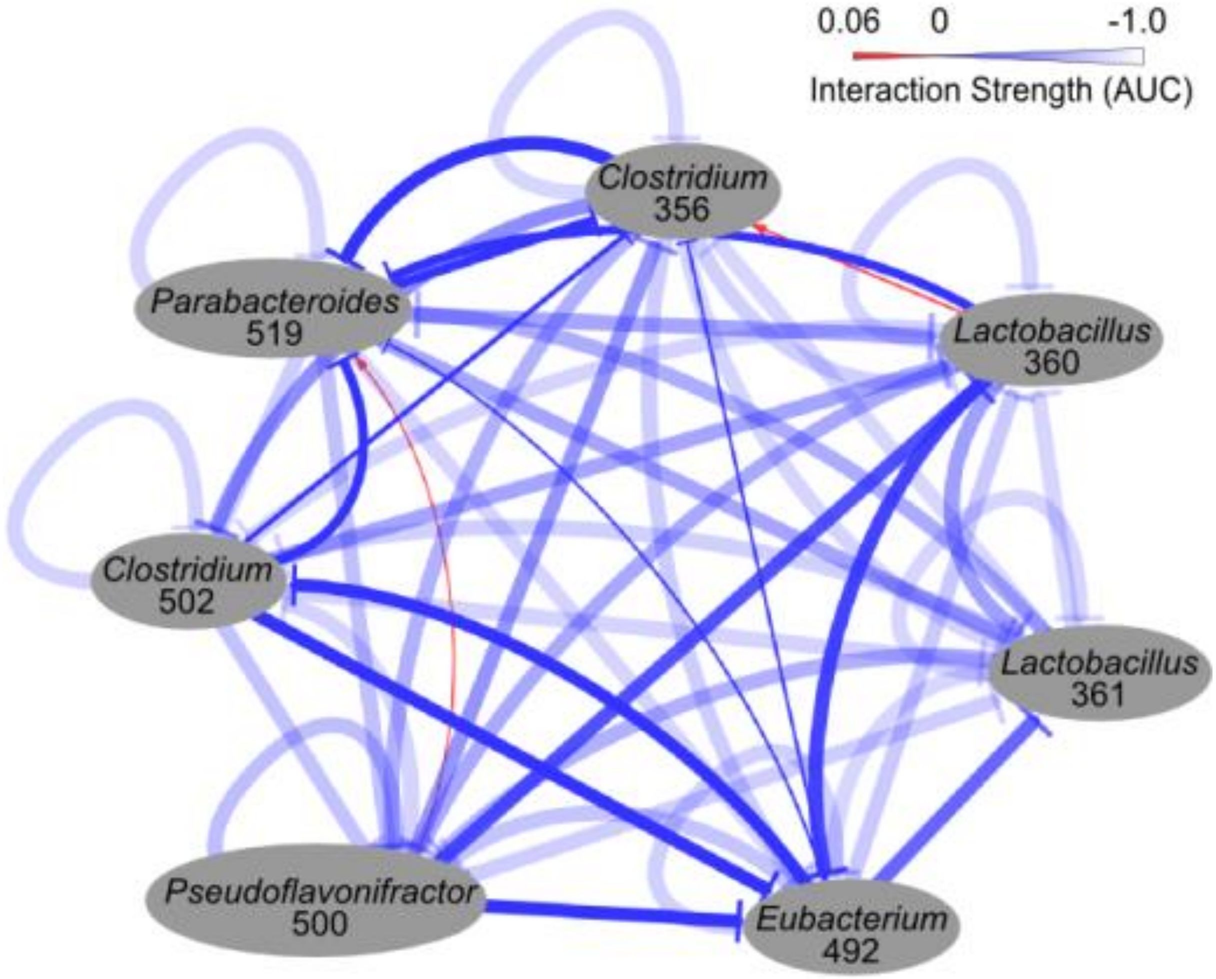

Supplemental Figure 8: Summary ASF interaction network. The interaction strength between each pair of species was calculated as fractional inhibition (see Figure 4A). For example, consider "AUC_alone" to be the area under the growth curve (AUC) of *Lactobacillus* 360 grown alone, and "AUC_356" to be the AUC for *Lactobacillus* 360 grown in spent356. The influence of *Clostridium* 356 on *Lactobacillus* 360 was calculated as (AUC_356 - AUC_alone) / AUC_alone. This results in a value close to -1 because *Lactobacillus* 360 is completely inhibited by spent media from *Clostridium* 356. Strong inhibitory relationships (i.e. -1) are shown by wide, faint, blue lines. Weaker relationships are shown by thin lines in darker color. Most relationships were very inhibitory under these experimental conditions, including all self-edges. Two relationships were weakly growth-promoting (thin, red lines).

Supplemental Table 1. NMR detection of expected compounds. Based on previous work defining the composition of LB, we composed a list of compounds expected to be present in the supplemented LB media. We gathered ^{1}H NMR spectra for fresh media samples and attempted to confirm the presence of these expected compounds. A subset of components expected to be in the media do not contain hydrogen bonds and are not detectable by ^{1}H NMR (such as Ca+, Cl-, Fe_2+, etc.). The majority of expected compounds are clearly detectable (30/44), while several are on the border of the detectable limit (6/44). A small group of compounds were not detected (8/44) either because they were at a concentration below the detectable limit, or a reference spectrum was not available.

**Expected Compound**	**NMR Detection**
Adenosine	Detected
AMP	Very low / border of detectable limit
CMP	Detected
Cytosine	Very low / border of detectable limit
Deoxyadenosine	Very low / border of detectable limit
Deoxycytidine	Difficult to identify this component
D-Glucose	Detected
Ethanol	Detected
Folate	Difficult to identify this component
Glycine	Detected
GMP	Detected
Guanosine	Detected
Hemin	No standard or reference spectra
Heme	No standard or reference spectra
Inosine	Detected
Lactose	Detected
L-Alanine	Detected
L-Arginine	Detected
L-Aspartate	Detected
L-Cysteine	Detected
L-Cystine	Detected
L-Glutamate	Detected
L-Histidine	Detected
Lipoate	Very low / border of detectable limit
L-Isoleucine	Detected
L-Leucine	Detected
L-Lysine	Detected
L-Methionine	Detected
L-Phenylalanine	Detected
L-Proline	Detected

L-Serine	Detected
L-Threonine	Detected
L-Tryptophan	Detected
L-Tyrosine	Detected
L-Valine	Detected
Niacin	Detected
Pyridoxal	Not detected–below detectable limit
Riboflavin	Very low / border of detectable limit
Shikimate	Not detected–below detectable limit
Thiamine phosphate	No standard or reference spectra
Thymidine	Not detected–below detectable limit
UMP	Very low / border of detectable limit
Uracil	Detected
Uridine	Detected

Supplemental Table 2. pH of ASF spent media. Each ASF member was grown in supplemented LB and the pH was measured using an Accumet pH meter.

Reps	ASF356	ASF360	ASF361	ASF492	ASF500	ASF502	ASF519
1	5.98	6.19	5.49	6.32	6.55	5.88	6.21
2	5.95	6.2	5.48	6.34	6.56	5.88	6.2
3	5.91	6.19	5.46	6.34	6.55	5.88	6.2
**Average**	**5.95**	**6.19**	**5.48**	**6.33**	**6.55**	**5.88**	**6.20**
**Stdev**	**0.029**	**0.005**	**0.012**	**0.009**	**0.005**	**0.000**	**0.005**

Supplemental Materials and Methods

Scanning Electron Microscopy

All ASF members excluding *Mucispirillum* ASF457 were cultured in BHI liquid medium in a 6-well plate containing an SEM stub coverslip. When growth was visible, cells were fixed for 30 min. with glutaraldehyde (2% by volume). Wells were rinsed 3 times for 5 min. each with 1xPBS. Samples were subjected to a graded ethanol dehydration, 10 min. each in 30, 50, 70, 80, 90, 100, 100% ethanol in water. The coverslips were further dehydrated for 10 min. in hexamethyldisilazane (HDMS) (Sigma). Finally, the coverslips were stuck to SEM stubs using the Phenom starter kit (Ted Pella, Redding, CA, USA) and sputter coated with gold using a SCD005 sputter coater (Bal-tec, Los Angeles, CA, USA). The final samples were imaged using a Sigma VP HD Field-emission SEM (Zeiss, Pleasanton, CA, USA).

Determining Colony Morphology

Agar plates were prepared using the supplemented LB medium described above (1.2% agar) except for *Mucispirillum*ASF457 which was grown on supplemented BHI (1.2% agar). ASF members were streaked to single colonies and grown for 70 hours (h) 37°C in the anaerobic chamber. Images were obtained using an EVOS XL digital inverted microscope (ThermoFisher Scientific, Waltham, MA, USA) with 2X objective.

Genomic Analysis and Comparison with Wild Murine Microbiota

Metagenomic data from the feces of 15 wild mice in a previous study (Wang et al. 2014) was used as a reference data set. Gene calls, performed using FragGeneScan (Rho et al. 2010), were downloaded from MG-RAST (Meyer et al. 2008) for the 15 mice specified at the W0 time point in Table S5 of (Wang et al. 2014) (MG-RAST project URL: http://metagenomics.anl.gov/?page=MetagenomeProject&project=5130). Gene calls were annotated with HMMER Version 3.1b2 (Eddy 1998), using bactNOG (144,498 protein sequences) from eggNOG version 4.1(Powell et al. 2014) as the profile hidden Markov models. For each

gene call, a non-supervised orthologous group (NOG) was assigned using the database target with the lowest e-value below 10^{-10}. Representative protein sequences for each ASF species were downloaded from GenBank (accession numbers: AQFQ00000000.1, AQFR00000000.1, AQFS00000000.1, AYGZ00000000.1, AQFT00000000.1, AYJP00000000.1, AQFU00000000.1, AQFV00000000.1) and searched against bactNOG for functional orthologs in the same manner as metagenomic gene calls. HMMER searches were performed on the University of Virginia high performance computing cluster.

To compare metagenome coverage by the ASF to coverage by random communities, species were drawn from the Firmicutes and Bacteroidetes phyla within bactNOG in a 6:2 ratio, respectively, to represent the most abundant phyla in the mouse gastrointestinal tract. Random communities of size 8, 16, 32, 64, and 128 were compared to the ASF for percent coverage of NOGs annotated in any metagenomic sample. This coverage was further sorted by sample frequency, where each NOG can occur in up to 15 metagenomic samples. NOGs containing functional annotations in more than one category were discarded during all portions of analysis (representing <1% of total annotations in any sample).

Media Preparation

Supplemented Brain-Heart Infusion: BHI base in power form (BD, Franklin Lakes, NJ, USA) was supplemented with yeast extract (5 grams/liter (g/l)), dissolved in deionized water and autoclaved at 121°C for 20 minutes (min.). After cooling, we added filter sterilized (0.22 micrometer (μm) pore size) solutions of vitamin K (2 microliters (μl)/l), hemin (5 milligrams (mg) /l), cysteine (0.5 g/l), and 5% each of newborn calf serum, horse serum, and sheep serum.

Media was equilibrated overnight in the anaerobic chamber before inoculation with ASF members.

Supplemented LB media for community studies

Reagent	Source	Catalogue number
LB base	Sigma	L3022 – 250G
L-cysteine	Sigma	30120 – 10G

Hemin	Sigma	H9030 – 1G
Vitamin K	Sigma	V3501 – 1G

Mineral salts solution (adapted from VPI Anaerobic Laboratory Manual)
Make the mineral salt solution in a volumetric flask and using RO-H_2O. Filter-sterilize (0.22µ) solution. Store at room temperature

Chemical	For 100 mL	For 500 mL
KH_2PO_4	0.6g	3g
$(NH_4)_2SO_4$	0.6g	3g
NaCl	1.2g	6g*
$MgSO_4{\cdot}7H_2O$	0.25g	1.25g
$CaCl_2{\cdot}7H_2O$	0.16g	0.8g

Supplements
Hemin (0.5 mg/mL): Dissolve 50mg in 1mL NaOH (1N) and bring to 100mL with RO-H_2O. Filter-sterilize (0.22µ). Store at 4°C. Add solution to fresh media before use – 1 mL/100 mL base.

Vitamin K (0.5%): Mix 100µL into 19.9mL 95% ethanol. Store at -20°C. Add solution to fresh media before use – 1 µL/10 mL base.

Lactose (5 mg/mL): Dissolve lactose in RO-H_2O. Filter-sterilize (0.22µ; 50mL conical). Store at RT (in anaerobic chamber). Add solution to fresh media before use – 10 µL/mL base.

Tween-20 (1 mg/mL): Dissolve Tween-20 in RO-H_2O. Filter-sterilize (0.22µ; 50mL conical). Store at RT (in anaerobic chamber). Add solution to fresh media before use – 10 µL/mL base.

Component	To prepare 250mL	To prepare 500mL	
LB base	7.5 g	15 g	Before Autoclave
Mineral salts sol'n	9.75 mL	19.5 mL	
L-cysteine	0.094 g	0.188 g	
Milli-Q (deionized) Water	232 mL	465 mL	
Hemin	3.75 mL	7.5 mL	After Autoclave
Vitamin K	3.75 µL	7.5 µL	
Lactose	3.75 mL	7.5 mL	
Tween-20	3.75 mL	7.5 mL	

Sterilize media (20 minutes on liquid cycle) and put into anaerobic chamber when cool. Before experiments, make fresh complete media by adding hemin, Vitamin K, lactose, and Tween-20 in the anaerobic chamber.

Growth Measurements

Growth curves were obtained for ASF members in the anaerobic chamber using four small plate readers measuring optical density at 870 nm (Jensen et al. 2015). The plate readers measure OD at 870 nm because this wavelength is not absorbed by common bacterial pigments (Jensen et al. 2015). Furthermore, because each LED pair is independent, the overall growth curve needs to be normalized to a common standard. Overnight liquid cultures of 10 ml were prepared for each ASF member: The entire volume of the overnight cultures were centrifuged at 8 000 rpm for 2 min. and the resulting pellets were resuspended in fresh liquid medium to produce a dense suspension of 0.75 ml. The optical density of the suspension was obtained on a Tecan (Männedorf, Switzerland) plate reader (600 nm). Liquid cultures were prepared in six-well plates with 6 ml per well, and inoculated (from the dense suspension) to a starting OD600 of 0.001. Each experimental condition was replicated four times. Each plate was covered with a Breath-Easy membrane (Sigma). The OD870 was tracked for 70 h. At the final time point, the OD600 of each well

was measured on the Tecan. The growth curves obtained at OD870 were normalized to the initial and final OD600 measurements. We chose to normalize to OD600 because it is a wavelength in common usage. For each well of the 6-well plate, growth curves from four independent LED pairs were averaged to produce a single growth curve per well. To determine the area under a growth curve (AUC), we applied trapezoidal numerical integration. The R (The R Foundation, Vienna, Austria) code for growth curve analysis is available in the open online repository.

pH Measurements

pH measurements were obtained with an Accumet Basic AB15 pH meter (Fisher Scientific, Hampton, NH, USA).

Determining Substrate Utilization and Byproduct Consumption with NMR Spectroscopy

Media (fresh or spent) samples of 2 ml were filter sterilized (0.22 μm pore size) and frozen at -80°C. Samples were prepared for ^{1}H NMR spectroscopy as described in (Dona et al. 2014). Samples were thawed at room temperature and centrifuged at 12 000 *g* at 4°C for 10 min., before 540 μl of supernatant was combined with 60 μl of buffer (pH 7.4; 1.5mM KH_2PO_4, 0.1% TSP (3-(trimethylsilyl)propionic-2,2,3,3-d4 acid sodium salt) in 100% D_2O) and transferred to a SampleJet NMR tube (Bruker BioSpin, Rheinstetten, Germany). Standard one-dimensional (1D) ^{1}H-NMR spectra with water pre-saturation were acquired at 300 K using a 600 MHz Avance III spectrometer (Bruker), equipped with a SampleJet autosampler (Bruker). A total of 32 scans were collected into 64 k data points for each sample. Spectra were automatically phased, baseline corrected and calibrated to the TSP resonance at $\delta^{1}H$ 0 in Topspin 3.1 software (Bruker).

The spectra were imported into Matlab R2014a (The Mathworks, Inc., Natick, MA, USA). Biologically irrelevant regions of the spectra were removed (TSP resonance at $\delta^{1}H$ 0 and residual water peak $\delta^{1}H$ 4.5-5.2) before peak alignment by recursive segment-wise peak alignment (RSPA) (Veselkov et al. 2009). The loadings of pairwise principal component analysis models, comparing blank media with the spent

media of each bacteria species, were used to identify metabolites generated or consumed in each experiment. The relevant regions of the spectra were integrated to calculate relative spectral intensities for each metabolite. Relative intensities in spent and double spent media were converted to z-scores with respect to metabolite abundances in fresh media. We defined significant abundance changes as those of magnitude greater than ±2 standard deviations from zero (zero being the metabolite abundance in fresh media). The peak integral data and associated R code for analysis and visualization is available in the online repository.

Classifying Instances of Emergent Metabolism

In this study, we define cases of "emergent metabolism" as any metabolic behavior which changes in the presence of another species. This broad definition can be divided into 6 categories:

1. ASF species only produced metabolite x when grown in fresh media
2. ASF species only consumed metabolite x when grown in spent media
3. ASF species switched from producing metabolite x (when grown in fresh media) to consuming metabolite x when grown in spent media
4. ASF species produced metabolite x only when grown in spent media
5. ASF species only consumed metabolite x when grown in fresh media
6. ASF species switched from consuming metabolite x (when grown in fresh media) to producing metabolite x when grown in spent media

We define "rare emergent metabolites" for each ASF species as metabolites which display emergent behavior (any of the 6 categories) in a single condition.

We identified all cases of emergent metabolism by comparing the metabolomics data from each single spent media sample to the double spent media samples for the same species. For example, when *Clostridium* ASF356 is grown in fresh media, it does not produce or consume methionine. When *Lactobacillus* ASF360 is grown in fresh media, is also does not produce or consume methionine. However, when *Clostridium* ASF356 is grown in the spent media from *Lactobacillus* ASF360, it does produce methionine. This would be an example of category 4, where *Clostridium* ASF356 produced

methionine only when grown in the spent media from another species. We identified and categorized all cases of emergent metabolism using a custom R script, which is available in an open online repository.

Correlation of NOG Presence/Absence and Metabolite Consumption/Production

NOG presence was represented as a binary vector with a 1 or 0 indicating the presence or absence of that NOG in each of the seven ASF species which grew. Metabolite changes were represented as a vector with elements for each species indicating consumption (-1), no change (0), or production (1) when that species was grown in fresh media. Consumption and production were defined as -2 or +2 standard deviations from the relative abundance in fresh media, respectively. We excluded 60 metabolic NOGs which were present in all ASF members and 12 metabolites which held the same value in all spent media samples, because correlations are undefined if variance of either variable is zero. The Spearman correlation was calculated between all pairs of 2 202 metabolic NOGs in the ASF with each of 85 metabolites.

Code and Data Availability

Our data and analysis scripts are available at the following repository:

http://mbi2gs.github.io/asf_characterization/.

Some large analysis output files and annotation files for metagenomic data are excluded due to file hosting size limitations, but are available upon request from the authors or can be generated using the indicated raw data, HMMer, associated eggNOG files, and scripts in the repository.

Supplemental Metabolomics Plots: For all experimental conditions, we plotted the relative changes in NMR peak integral (z-score with respect fresh media) of each known metabolite in each individual replicate. Gray points with black boxes originate from the main data set. Red points and boxes originate from a second, independent set of biological replicates. The z-scores for the second data set were calculated using the standard deviations from the first data set, to facilitate comparisons between the two. Plots are labeled as "ASF-Species-in-Media-ID". There are eight plots for each species (one resulting from growth in fresh media, one from growth in its own spent media, and six resulting from growth in the spent media of the other ASF members).

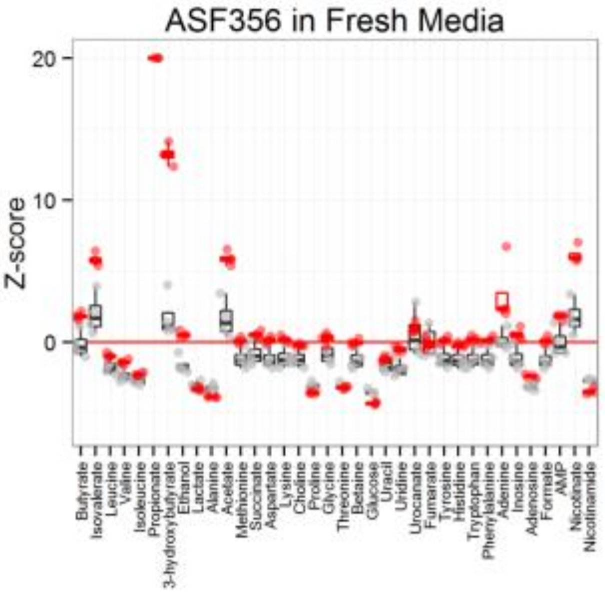

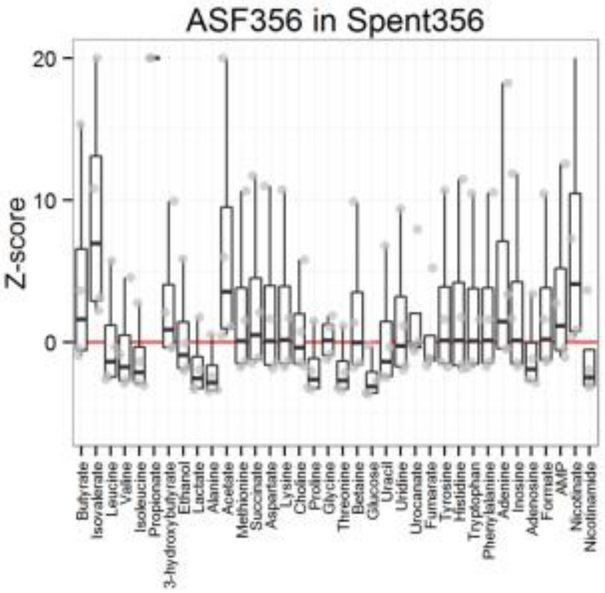

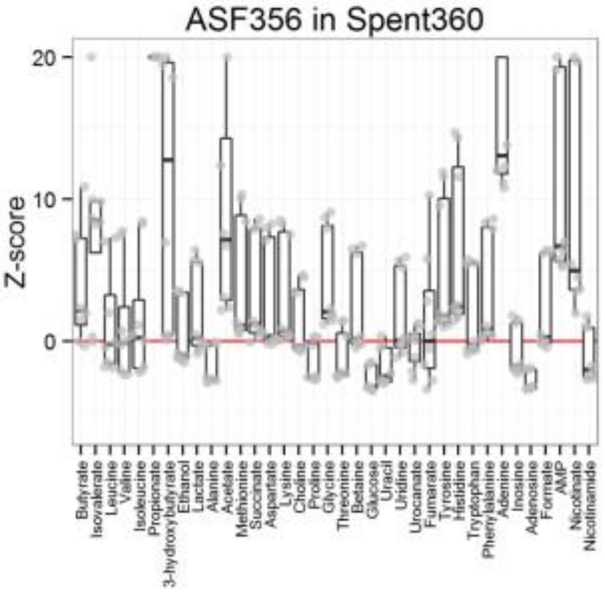

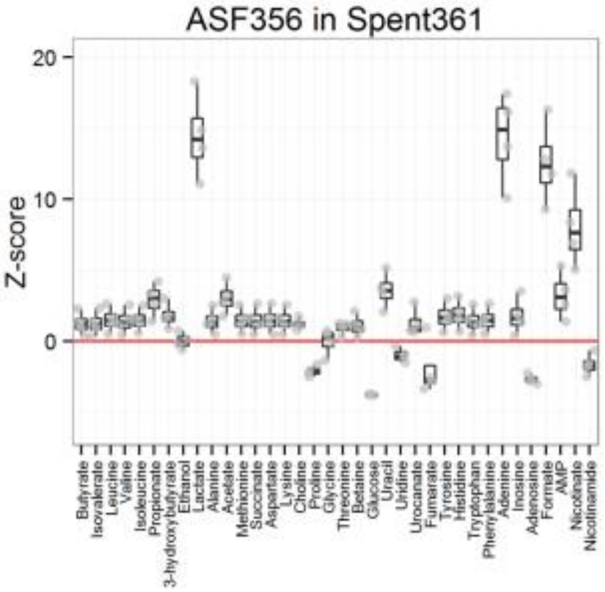

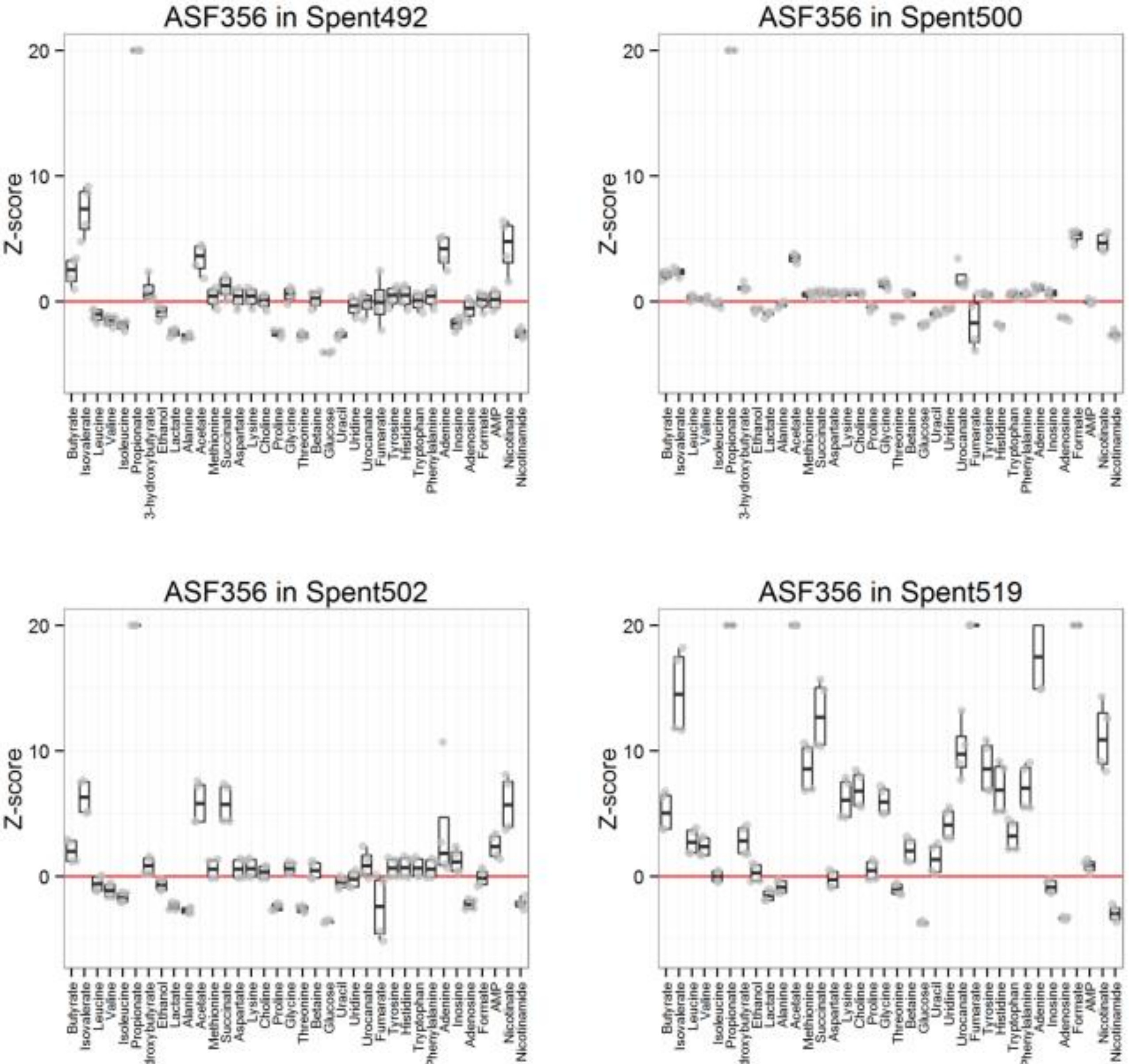
ASF356 in Spent492
ASF356 in Spent500
ASF356 in Spent502
ASF356 in Spent519
Z-score
20
10
0
Butyrate
Isovalerate
Leucine
Valine
Isoleucine
Propionate
3-hydroxybutyrate
Ethanol
Lactate
Alanine
Acetate
Methionine
Succinate
Aspartate
Lysine
Choline
Proline
Glycine
Threonine
Betaine
Glucose
Uracil
Uridine
Urocanate
Fumarate
Tyrosine
Histidine
Tryptophan
Phenylalanine
Adenine
Inosine
Adenosine
Formate
AMP
Nicotinate
Nicotinamide

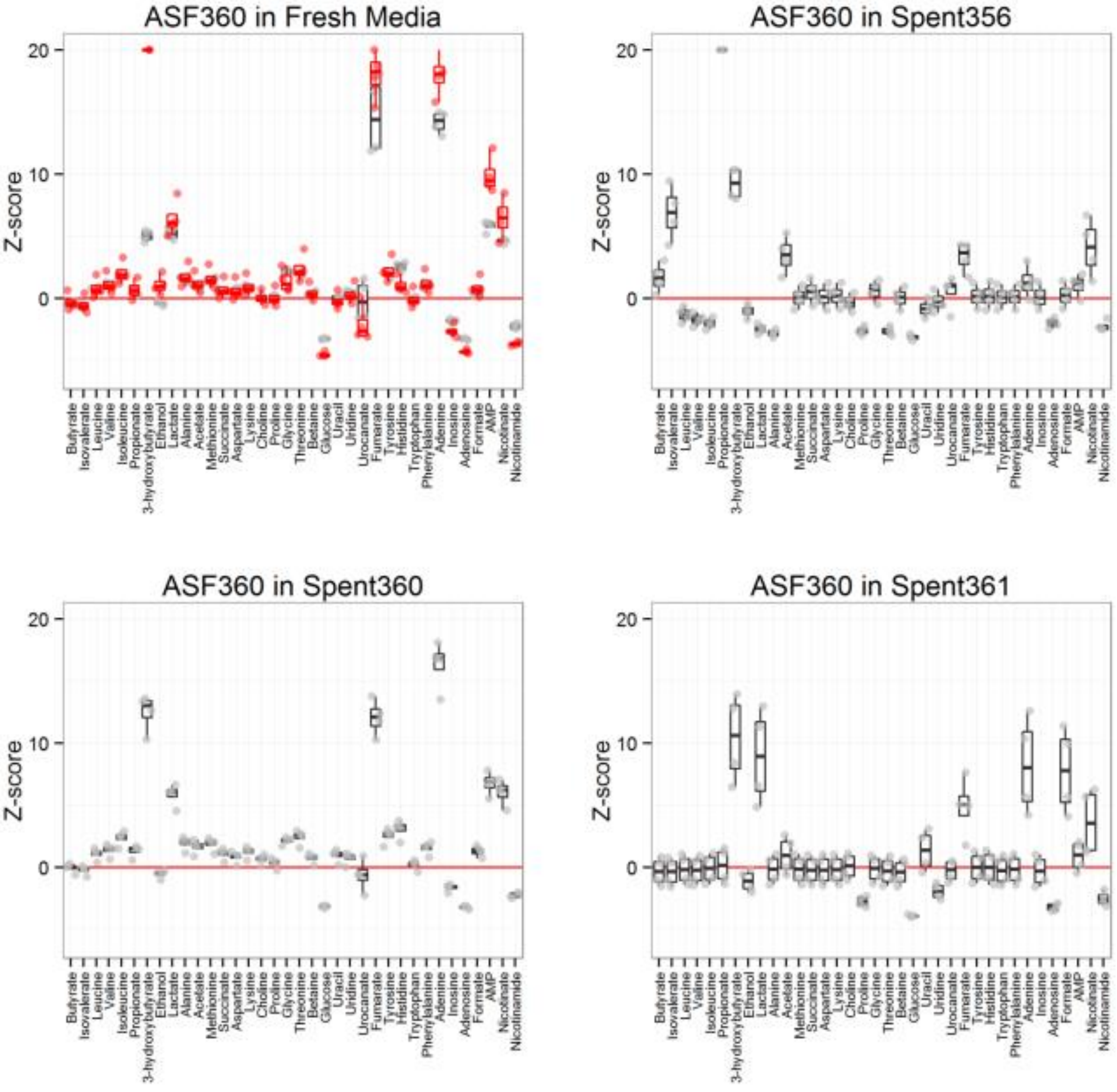
ASF360 in Fresh Media
Z-score
20
10
0
Butyrate
Isovalerate
Leucine
Valine
Isoleucine
Propionate
3-hydroxybutyrate
Ethanol
Lactate
Alanine
Acetate
Methionine
Succinate
Aspartate
Lysine
Choline
Proline
Glycine
Threonine
Betaine
Glucose
Uracil
Uridine
Urocanate
Fumarate
Tyrosine
Histidine
Tryptophan
Phenylalanine
Adenine
Inosine
Adenosine
Formate
AMP
Nicotinate
Nicotinamide
ASF360 in Spent356
Z-score
20
10
0
Butyrate
Isovalerate
Leucine
Valine
Isoleucine
Propionate
3-hydroxybutyrate
Ethanol
Lactate
Alanine
Acetate
Methionine
Succinate
Aspartate
Lysine
Choline
Proline
Glycine
Threonine
Betaine
Glucose
Uracil
Uridine
Urocanate
Fumarate
Tyrosine
Histidine
Tryptophan
Phenylalanine
Adenine
Inosine
Adenosine
Formate
AMP
Nicotinate
Nicotinamide
ASF360 in Spent360
Z-score
20
10
0
Butyrate
Isovalerate
Leucine
Valine
Isoleucine
Propionate
3-hydroxybutyrate
Ethanol
Lactate
Alanine
Acetate
Methionine
Succinate
Aspartate
Lysine
Choline
Proline
Glycine
Threonine
Betaine
Glucose
Uracil
Uridine
Urocanate
Fumarate
Tyrosine
Histidine
Tryptophan
Phenylalanine
Adenine
Inosine
Adenosine
Formate
AMP
Nicotinate
Nicotinamide
ASF360 in Spent361
Z-score
20
10
0
Butyrate
Isovalerate
Leucine
Valine
Isoleucine
Propionate
3-hydroxybutyrate
Ethanol
Lactate
Alanine
Acetate
Methionine
Succinate
Aspartate
Lysine
Choline
Proline
Glycine
Threonine
Betaine
Glucose
Uracil
Uridine
Urocanate
Fumarate
Tyrosine
Histidine
Tryptophan
Phenylalanine
Adenine
Inosine
Adenosine
Formate
AMP
Nicotinate
Nicotinamide

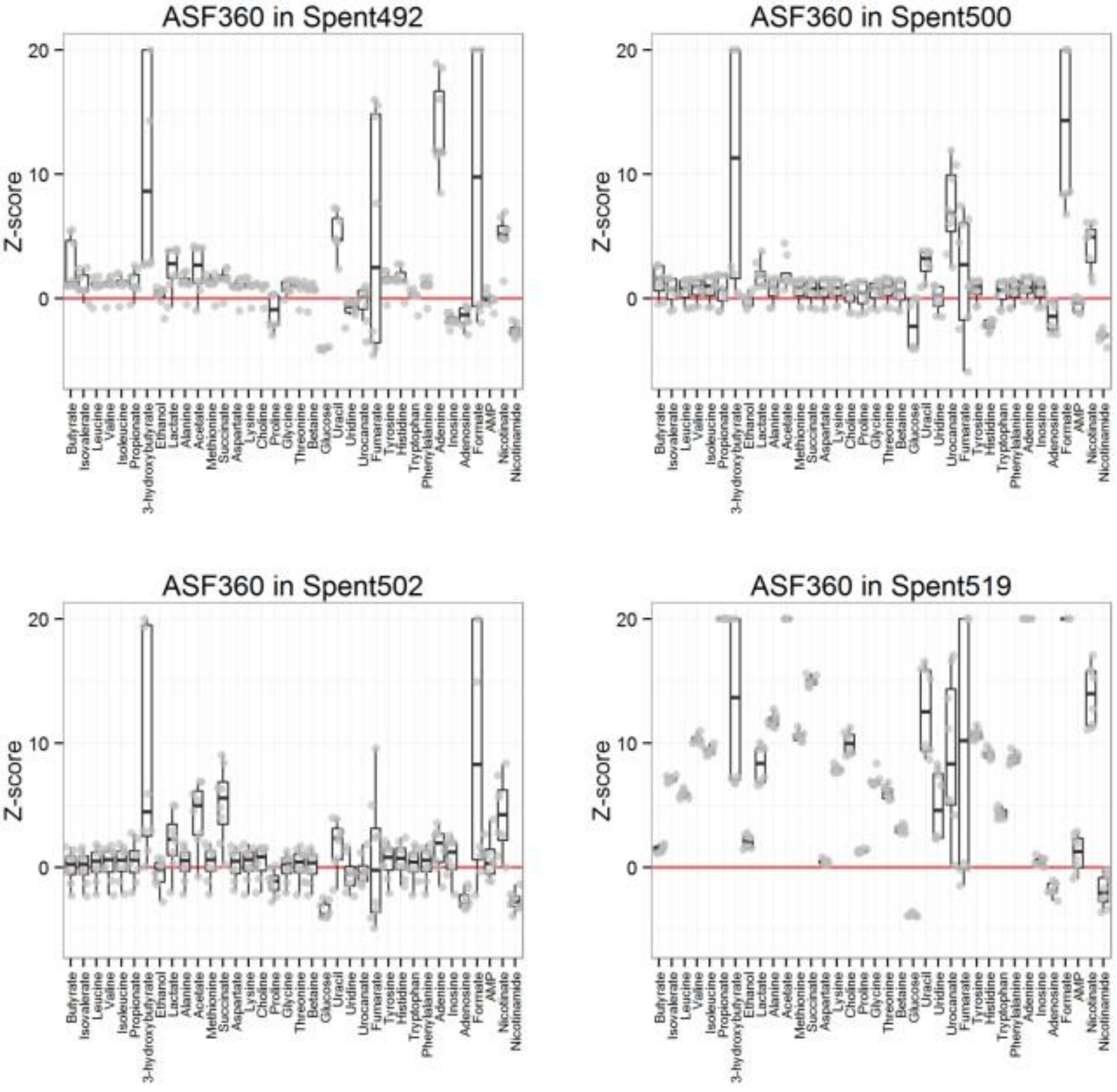

ASF360 in Spent492
ASF360 in Spent500
ASF360 in Spent502
ASF360 in Spent519
Z-score
20
10
0
Butyrate
Isovalerate
Leucine
Valine
Isoleucine
Propionate
3-hydroxybutyrate
Ethanol
Lactate
Alanine
Acetate
Methionine
Succinate
Aspartate
Lysine
Choline
Proline
Glycine
Threonine
Betaine
Glucose
Uracil
Uridine
Urocanate
Fumarate
Tyrosine
Histidine
Tryptophan
Phenylalanine
Adenine
Inosine
Adenosine
Formate
AMP
Nicotinate
Nicotinamide

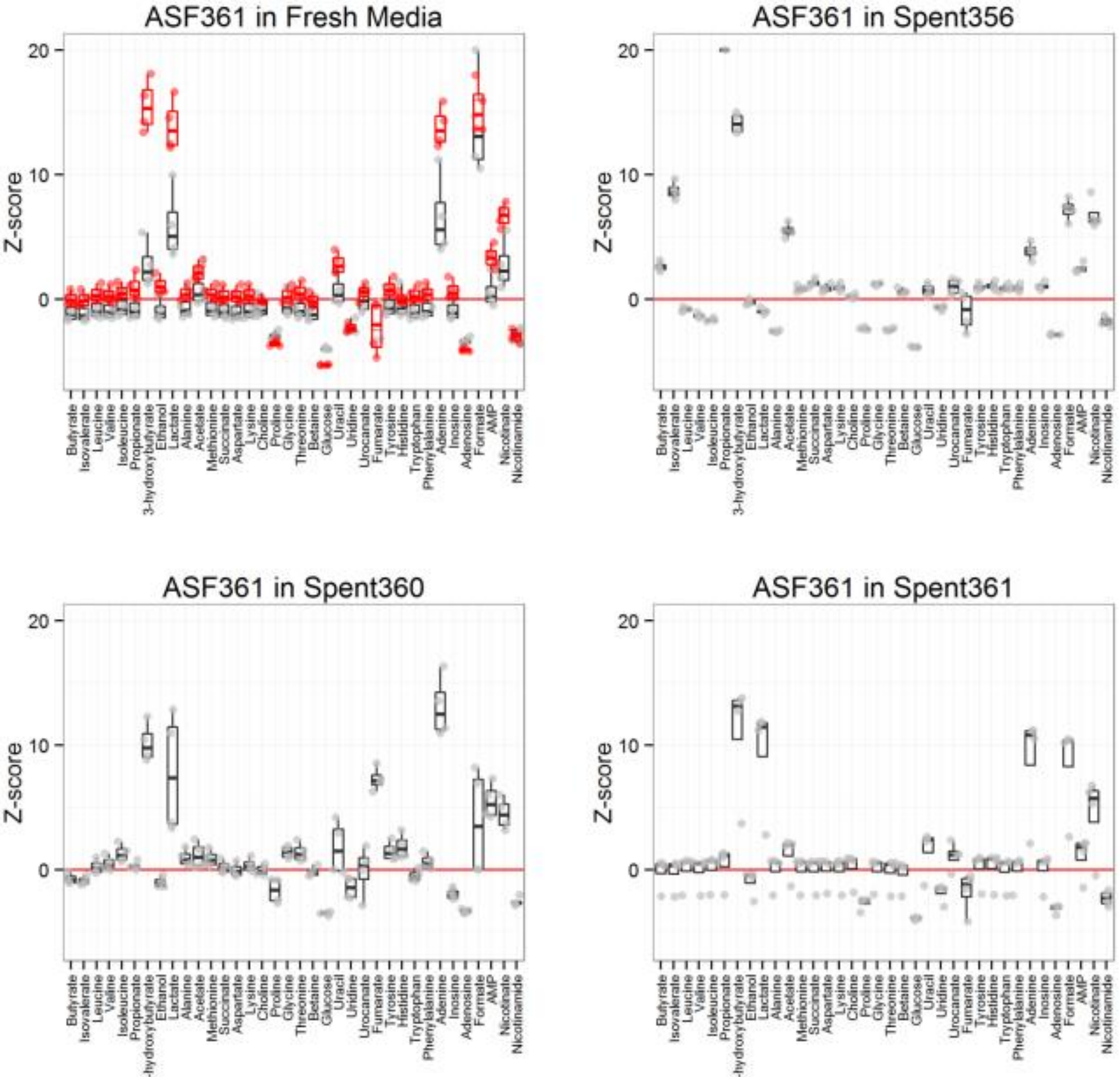
ASF361 in Fresh Media
ASF361 in Spent356
ASF361 in Spent360
ASF361 in Spent361
Z-score
20
10
0
Butyrate
Isovalerate
Leucine
Valine
Isoleucine
Propionate
3-hydroxybutyrate
Ethanol
Lactate
Alanine
Acetate
Methionine
Succinate
Aspartate
Lysine
Choline
Proline
Glycine
Threonine
Betaine
Glucose
Uracil
Uridine
Urocanate
Fumarate
Tyrosine
Histidine
Tryptophan
Phenylalanine
Adenine
Inosine
Adenosine
Formate
AMP
Nicotinate
Nicotinamide

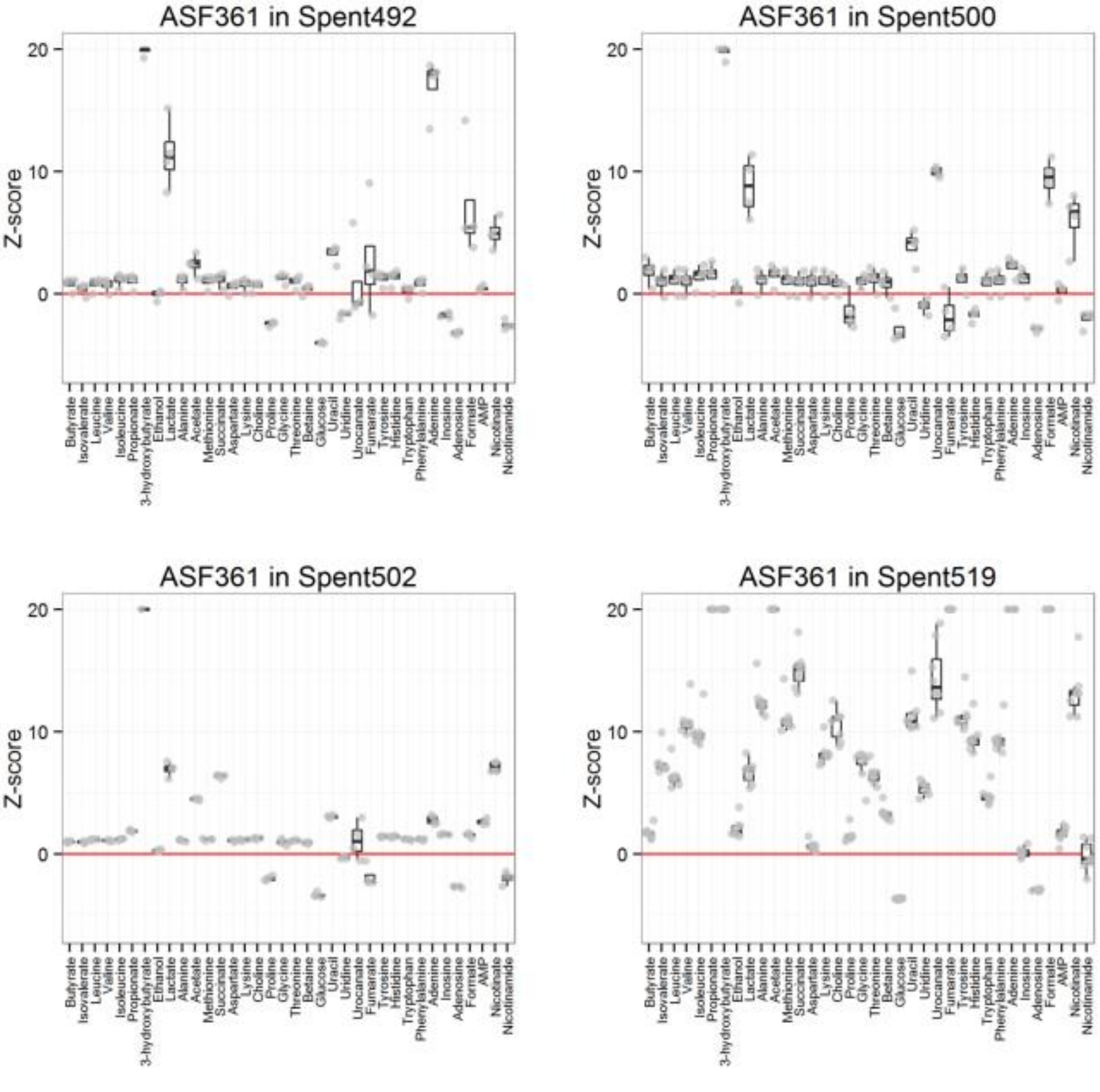
ASF361 in Spent492
ASF361 in Spent500
ASF361 in Spent502
ASF361 in Spent519
Z-score
20
10
0

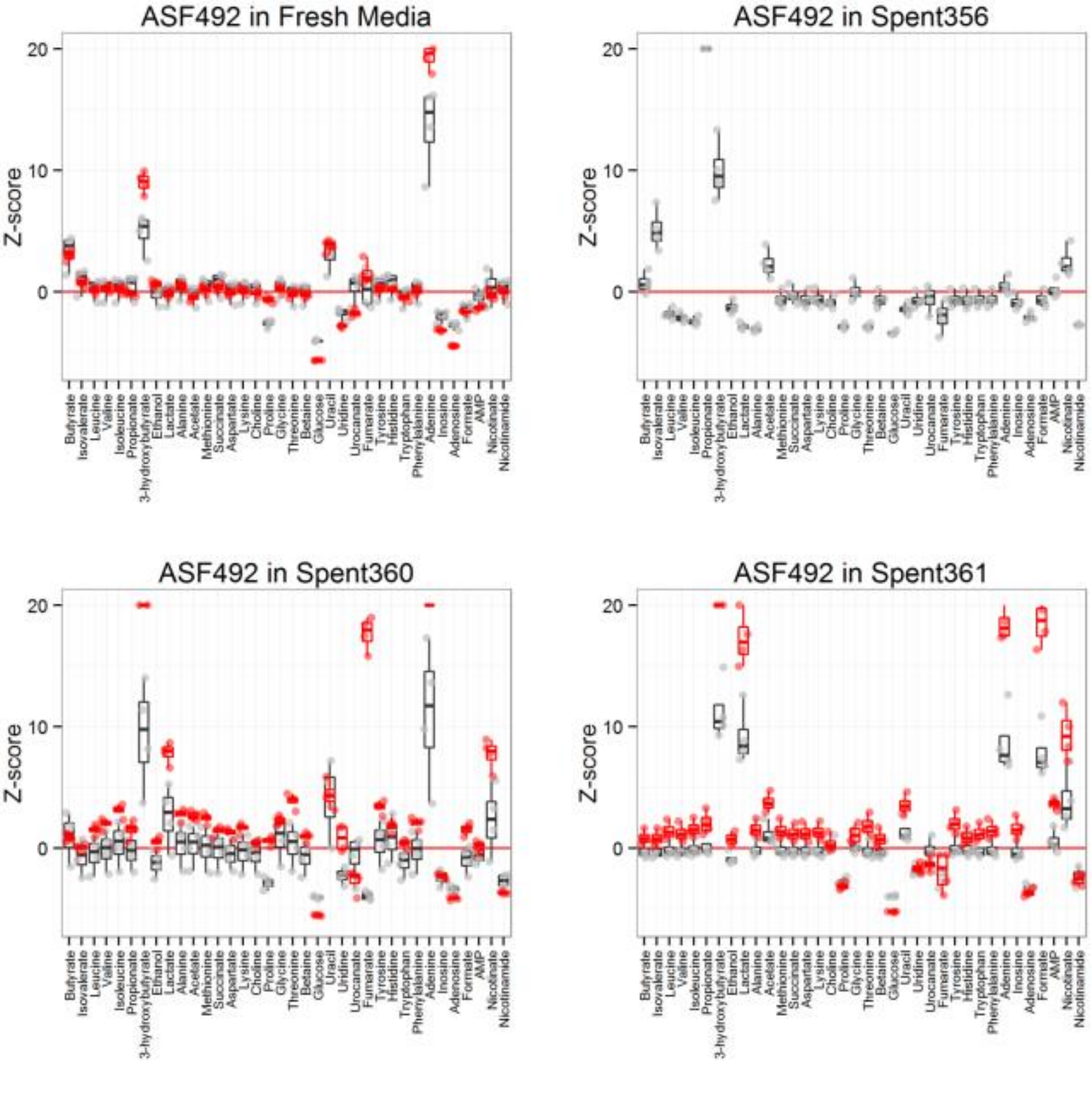
ASF492 in Fresh Media
ASF492 in Spent356
ASF492 in Spent360
ASF492 in Spent361
Z-score
0
10
20
Butyrate
Isovalerate
Leucine
Valine
Isoleucine
Propionate
3-hydroxybutyrate
Ethanol
Lactate
Alanine
Acetate
Methionine
Succinate
Aspartate
Lysine
Choline
Proline
Glycine
Threonine
Betaine
Glucose
Uracil
Uridine
Urocanate
Fumarate
Tyrosine
Histidine
Tryptophan
Phenylalanine
Adenine
Inosine
Adenosine
Formate
AMP
Nicotinate
Nicotinamide

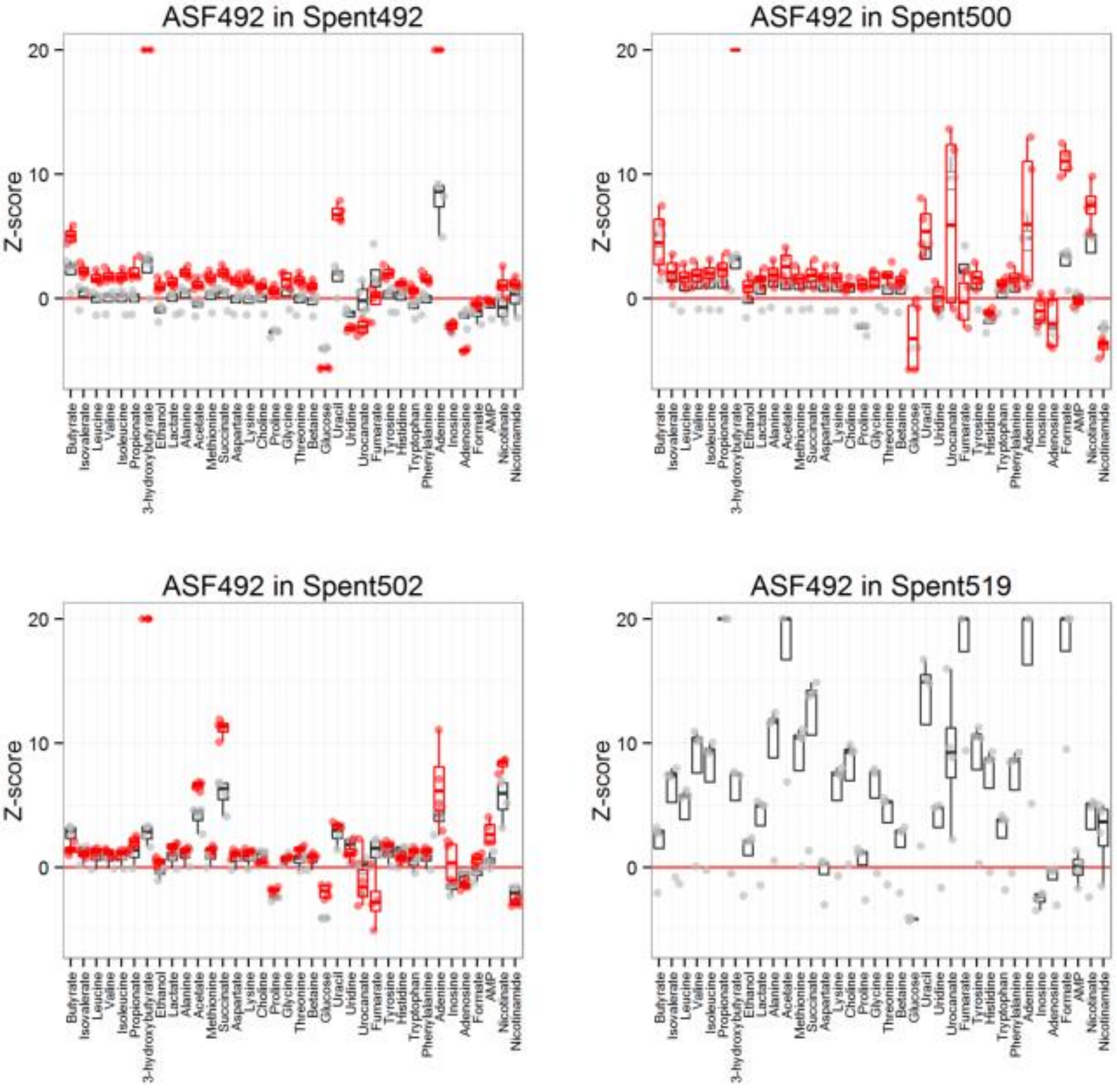
ASF492 in Spent492
Z-score
20
10
0
Butyrate
Isovalerate
Leucine
Valine
Isoleucine
Propionate
3-hydroxybutyrate
Ethanol
Lactate
Alanine
Acetate
Methionine
Succinate
Aspartate
Lysine
Choline
Proline
Glycine
Threonine
Betaine
Glucose
Uracil
Uridine
Urocanate
Fumarate
Tyrosine
Histidine
Tryptophan
Phenylalanine
Adenine
Inosine
Adenosine
Formate
AMP
Nicotinate
Nicotinamide
ASF492 in Spent500
Z-score
20
10
0
Butyrate
Isovalerate
Leucine
Valine
Isoleucine
Propionate
3-hydroxybutyrate
Ethanol
Lactate
Alanine
Acetate
Methionine
Succinate
Aspartate
Lysine
Choline
Proline
Glycine
Threonine
Betaine
Glucose
Uracil
Uridine
Urocanate
Fumarate
Tyrosine
Histidine
Tryptophan
Phenylalanine
Adenine
Inosine
Adenosine
Formate
AMP
Nicotinate
Nicotinamide
ASF492 in Spent502
Z-score
20
10
0
Butyrate
Isovalerate
Leucine
Valine
Isoleucine
Propionate
3-hydroxybutyrate
Ethanol
Lactate
Alanine
Acetate
Methionine
Succinate
Aspartate
Lysine
Choline
Proline
Glycine
Threonine
Betaine
Glucose
Uracil
Uridine
Urocanate
Fumarate
Tyrosine
Histidine
Tryptophan
Phenylalanine
Adenine
Inosine
Adenosine
Formate
AMP
Nicotinate
Nicotinamide
ASF492 in Spent519
Z-score
20
10
0
Butyrate
Isovalerate
Leucine
Valine
Isoleucine
Propionate
3-hydroxybutyrate
Ethanol
Lactate
Alanine
Acetate
Methionine
Succinate
Aspartate
Lysine
Choline
Proline
Glycine
Threonine
Betaine
Glucose
Uracil
Uridine
Urocanate
Fumarate
Tyrosine
Histidine
Tryptophan
Phenylalanine
Adenine
Inosine
Adenosine
Formate
AMP
Nicotinate
Nicotinamide

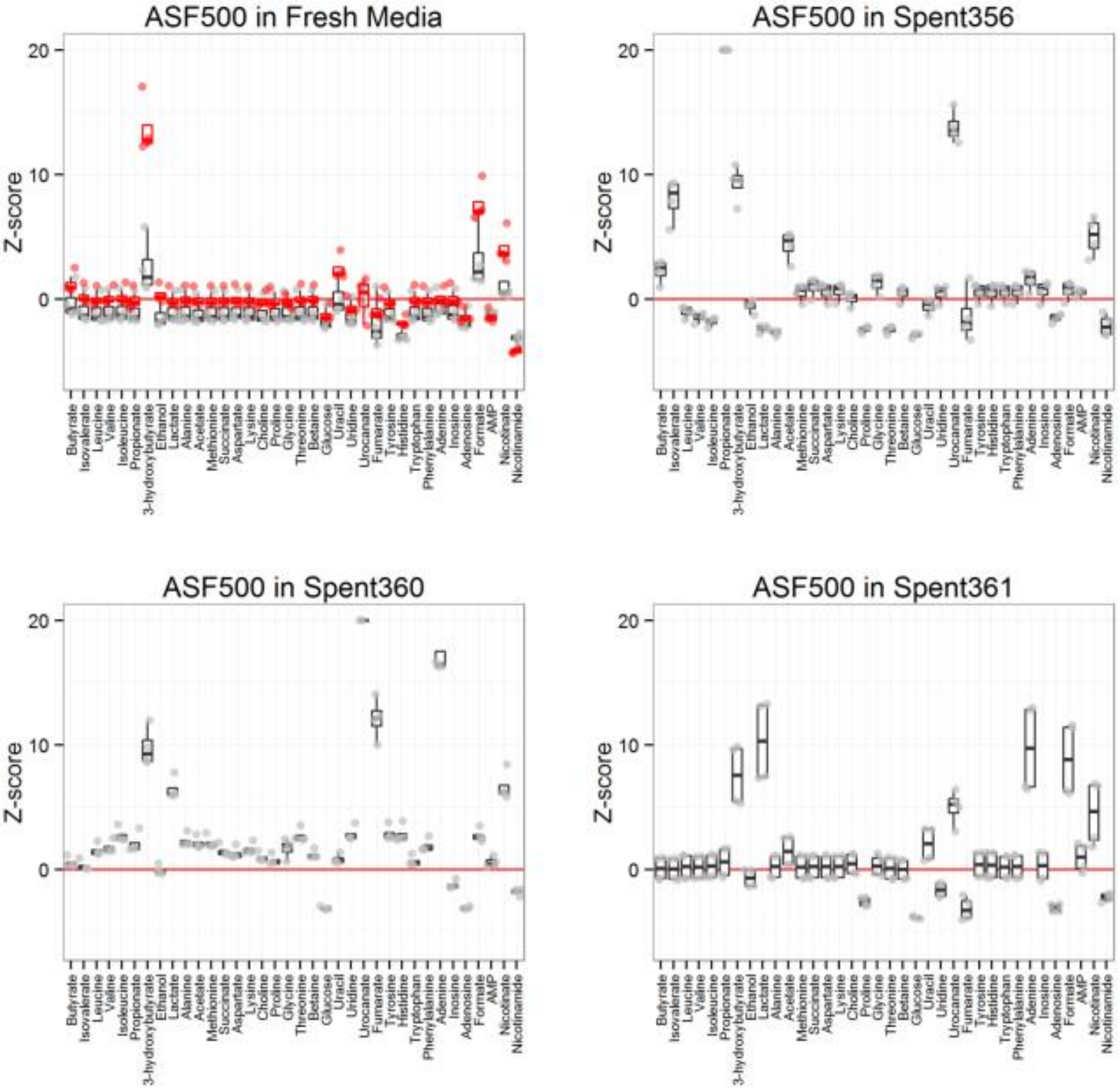

ASF500 in Fresh Media
Z-score
20
10
0
Butyrate
Isovalerate
Leucine
Valine
Isoleucine
Propionate
3-hydroxybutyrate
Ethanol
Lactate
Alanine
Acetate
Methionine
Succinate
Aspartate
Lysine
Choline
Proline
Glycine
Threonine
Betaine
Glucose
Uracil
Uridine
Urocanate
Fumarate
Tyrosine
Histidine
Tryptophan
Phenylalanine
Adenine
Inosine
Adenosine
Formate
AMP
Nicotinate
Nicotinamide
ASF500 in Spent356
Z-score
20
10
0
Butyrate
Isovalerate
Leucine
Valine
Isoleucine
Propionate
3-hydroxybutyrate
Ethanol
Lactate
Alanine
Acetate
Methionine
Succinate
Aspartate
Lysine
Choline
Proline
Glycine
Threonine
Betaine
Glucose
Uracil
Uridine
Urocanate
Fumarate
Tyrosine
Histidine
Tryptophan
Phenylalanine
Adenine
Inosine
Adenosine
Formate
AMP
Nicotinate
Nicotinamide
ASF500 in Spent360
Z-score
20
10
0
Butyrate
Isovalerate
Leucine
Valine
Isoleucine
Propionate
3-hydroxybutyrate
Ethanol
Lactate
Alanine
Acetate
Methionine
Succinate
Aspartate
Lysine
Choline
Proline
Glycine
Threonine
Betaine
Glucose
Uracil
Uridine
Urocanate
Fumarate
Tyrosine
Histidine
Tryptophan
Phenylalanine
Adenine
Inosine
Adenosine
Formate
AMP
Nicotinate
Nicotinamide
ASF500 in Spent361
Z-score
20
10
0
Butyrate
Isovalerate
Leucine
Valine
Isoleucine
Propionate
3-hydroxybutyrate
Ethanol
Lactate
Alanine
Acetate
Methionine
Succinate
Aspartate
Lysine
Choline
Proline
Glycine
Threonine
Betaine
Glucose
Uracil
Uridine
Urocanate
Fumarate
Tyrosine
Histidine
Tryptophan
Phenylalanine
Adenine
Inosine
Adenosine
Formate
AMP
Nicotinate
Nicotinamide

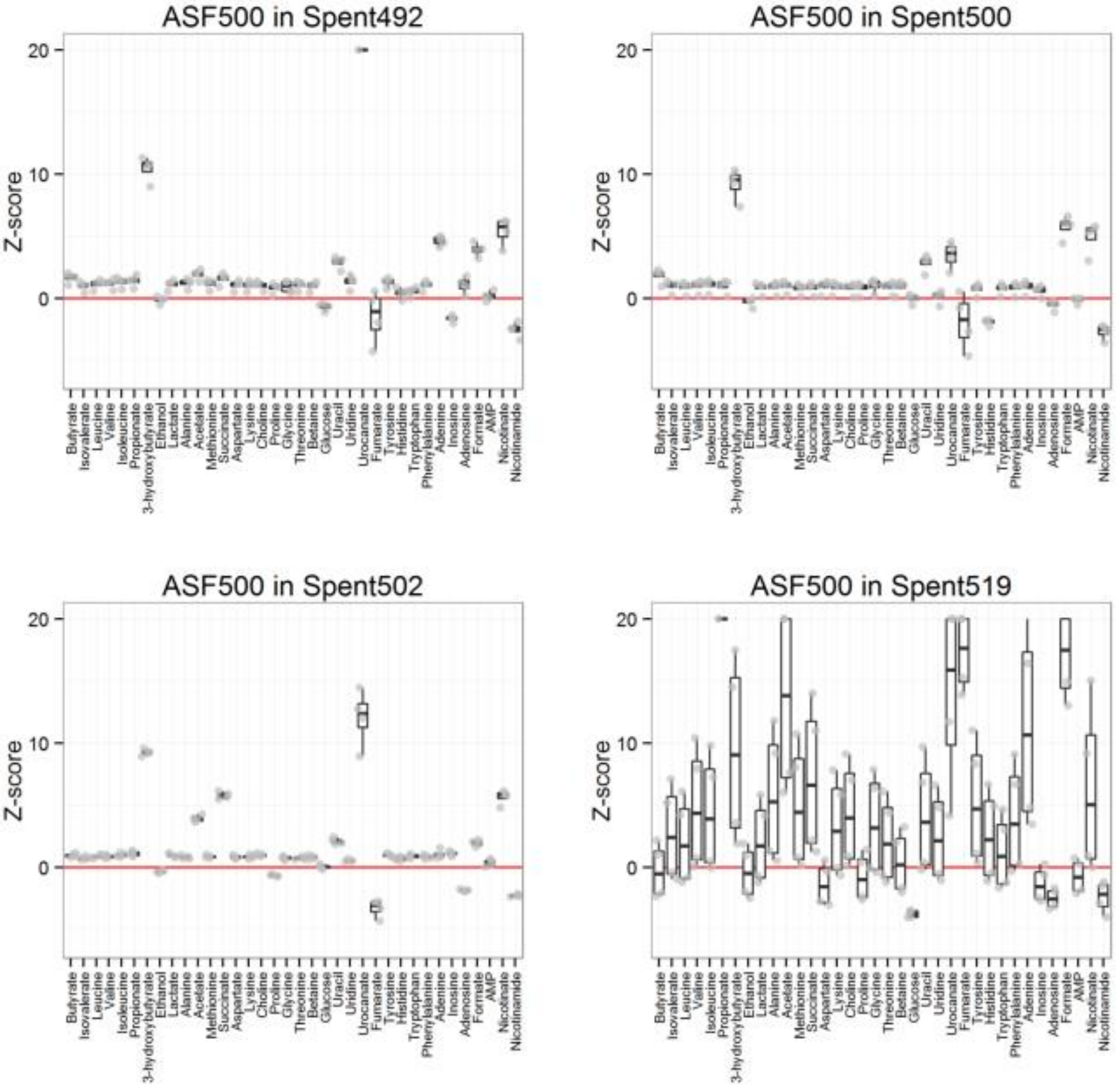

ASF500 in Spent492
ASF500 in Spent500
ASF500 in Spent502
ASF500 in Spent519
Z-score
20
10
0
Butyrate
Isovalerate
Leucine
Valine
Isoleucine
Propionate
3-hydroxybutyrate
Ethanol
Lactate
Alanine
Acetate
Methionine
Succinate
Aspartate
Lysine
Choline
Proline
Glycine
Threonine
Betaine
Glucose
Uracil
Uridine
Urocanate
Fumarate
Tyrosine
Histidine
Tryptophan
Phenylalanine
Adenine
Inosine
Adenosine
Formate
AMP
Nicotinate
Nicotinamide

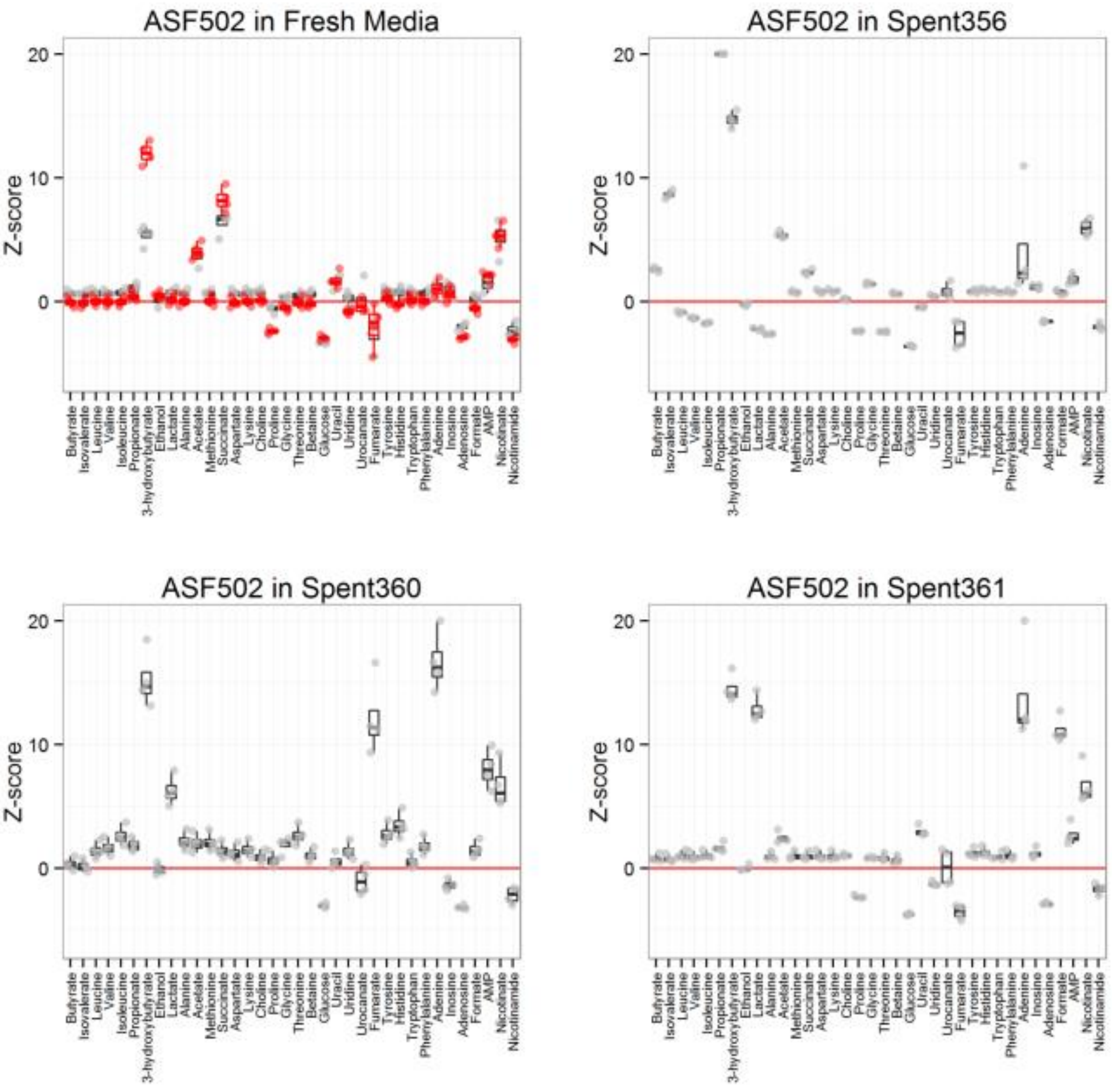

ASF502 in Fresh Media
ASF502 in Spent356
ASF502 in Spent360
ASF502 in Spent361
Z-score
20
10
0
Butyrate
Isovalerate
Leucine
Valine
Isoleucine
Propionate
3-hydroxybutyrate
Ethanol
Lactate
Alanine
Acetate
Methionine
Succinate
Aspartate
Lysine
Choline
Proline
Glycine
Threonine
Betaine
Glucose
Uracil
Uridine
Urocanate
Fumarate
Tyrosine
Histidine
Tryptophan
Phenylalanine
Adenine
Inosine
Adenosine
Formate
AMP
Nicotinate
Nicotinamide

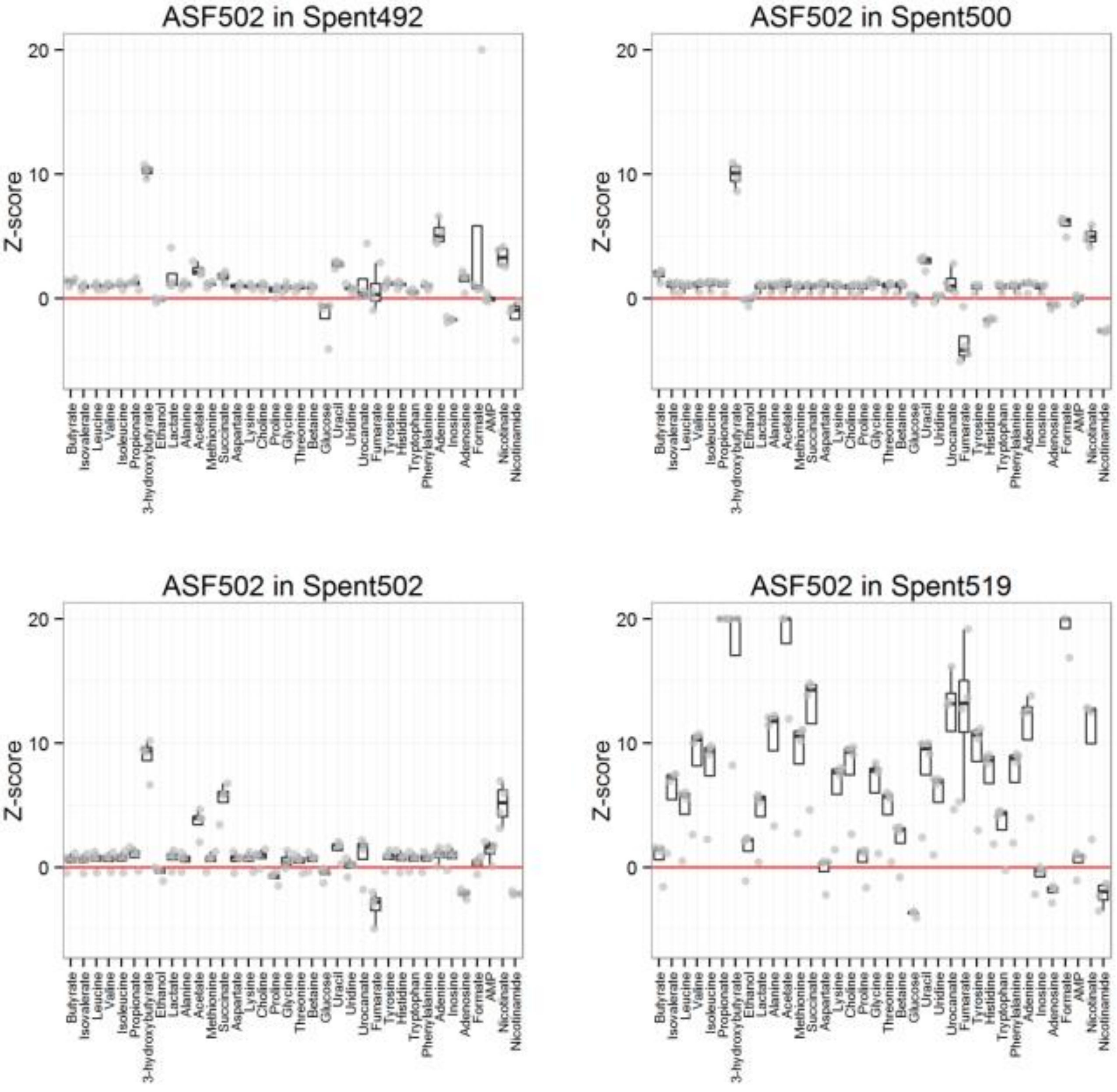

ASF502 in Spent492
ASF502 in Spent500
ASF502 in Spent502
ASF502 in Spent519
Z-score
20
10
0
Butyrate
Isovalerate
Leucine
Valine
Isoleucine
Propionate
3-hydroxybutyrate
Ethanol
Lactate
Alanine
Acetate
Methionine
Succinate
Aspartate
Lysine
Choline
Proline
Glycine
Threonine
Betaine
Glucose
Uracil
Uridine
Urocanate
Fumarate
Tyrosine
Histidine
Tryptophan
Phenylalanine
Adenine
Inosine
Adenosine
Formate
AMP
Nicotinate
Nicotinamide

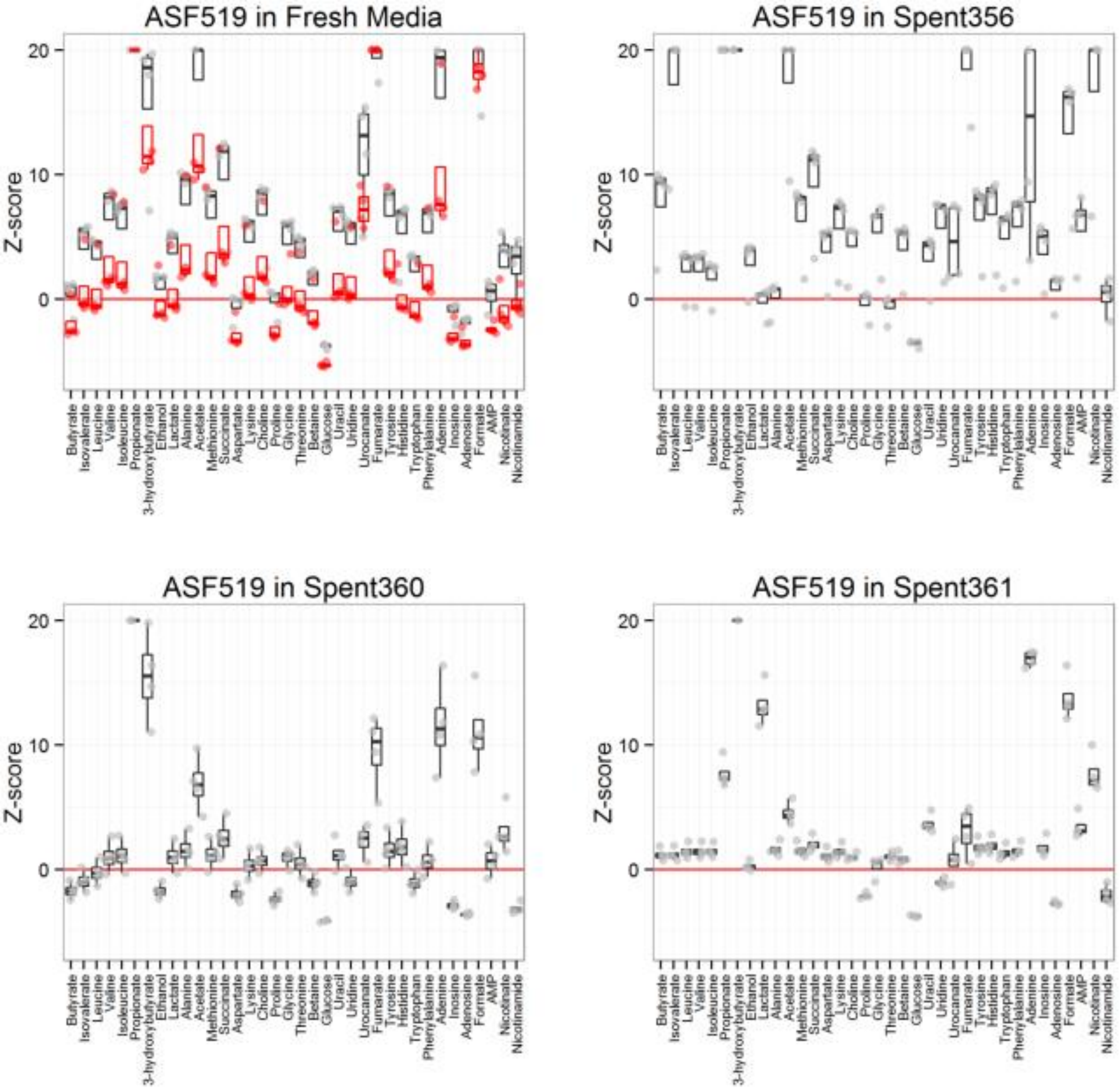

ASF519 in Fresh Media
Z-score
20
10
0
Butyrate
Isovalerate
Leucine
Valine
Isoleucine
Propionate
3-hydroxybutyrate
Ethanol
Lactate
Alanine
Acetate
Methionine
Succinate
Aspartate
Lysine
Choline
Proline
Glycine
Threonine
Betaine
Glucose
Uracil
Uridine
Urocanate
Fumarate
Tyrosine
Histidine
Tryptophan
Phenylalanine
Adenine
Inosine
Adenosine
Formate
AMP
Nicotinate
Nicotinamide
ASF519 in Spent356
Z-score
20
10
0
Butyrate
Isovalerate
Leucine
Valine
Isoleucine
Propionate
3-hydroxybutyrate
Ethanol
Lactate
Alanine
Acetate
Methionine
Succinate
Aspartate
Lysine
Choline
Proline
Glycine
Threonine
Betaine
Glucose
Uracil
Uridine
Urocanate
Fumarate
Tyrosine
Histidine
Tryptophan
Phenylalanine
Adenine
Inosine
Adenosine
Formate
AMP
Nicotinate
Nicotinamide
ASF519 in Spent360
Z-score
20
10
0
Butyrate
Isovalerate
Leucine
Valine
Isoleucine
Propionate
3-hydroxybutyrate
Ethanol
Lactate
Alanine
Acetate
Methionine
Succinate
Aspartate
Lysine
Choline
Proline
Glycine
Threonine
Betaine
Glucose
Uracil
Uridine
Urocanate
Fumarate
Tyrosine
Histidine
Tryptophan
Phenylalanine
Adenine
Inosine
Adenosine
Formate
AMP
Nicotinate
Nicotinamide
ASF519 in Spent361
Z-score
20
10
0
Butyrate
Isovalerate
Leucine
Valine
Isoleucine
Propionate
3-hydroxybutyrate
Ethanol
Lactate
Alanine
Acetate
Methionine
Succinate
Aspartate
Lysine
Choline
Proline
Glycine
Threonine
Betaine
Glucose
Uracil
Uridine
Urocanate
Fumarate
Tyrosine
Histidine
Tryptophan
Phenylalanine
Adenine
Inosine
Adenosine
Formate
AMP
Nicotinate
Nicotinamide

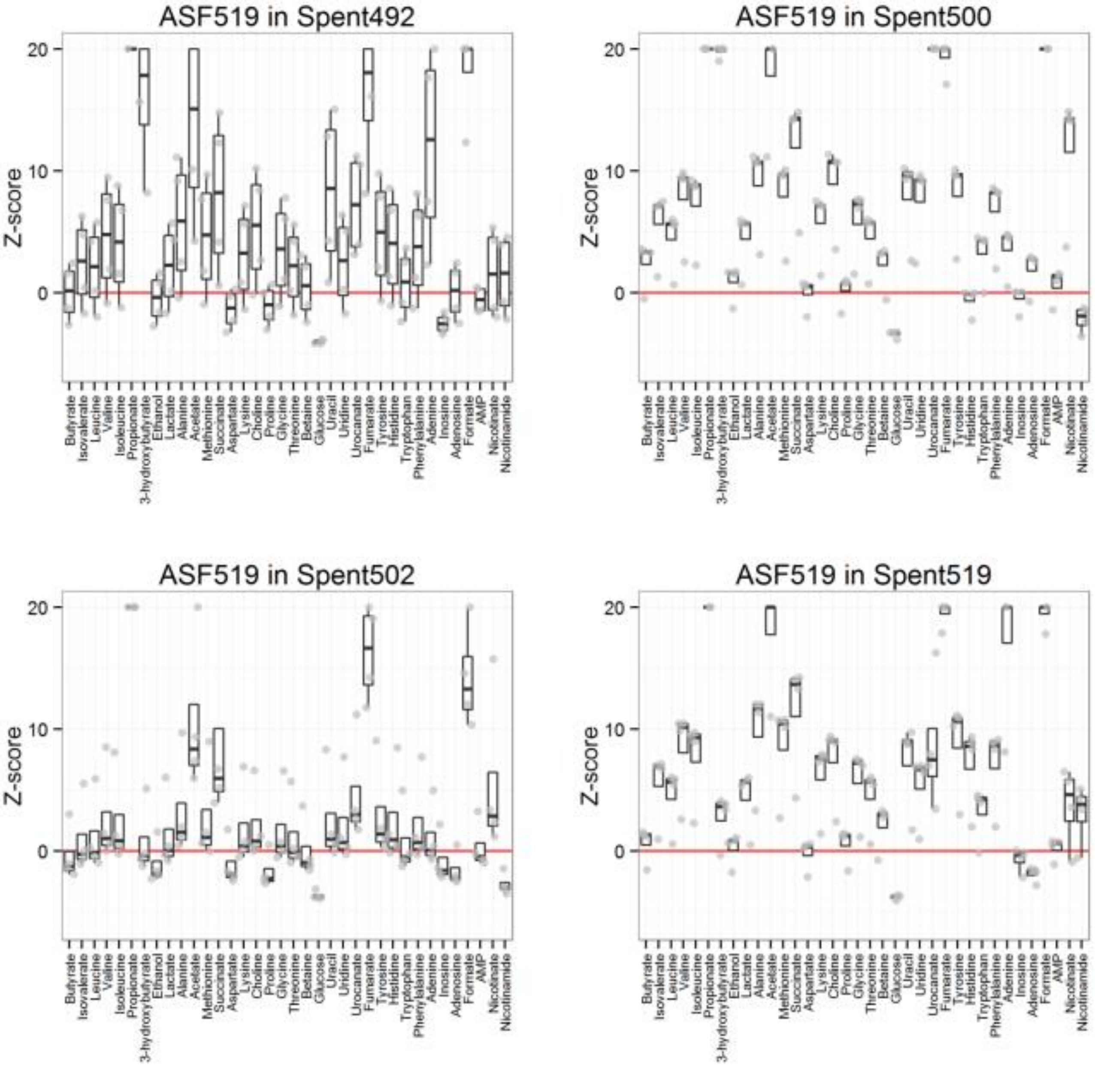
ASF519 in Spent492
ASF519 in Spent500
ASF519 in Spent502
ASF519 in Spent519
Z-score
20
10
0
Butyrate
Isovalerate
Leucine
Valine
Isoleucine
Propionate
3-hydroxybutyrate
Ethanol
Lactate
Alanine
Acetate
Methionine
Succinate
Aspartate
Lysine
Choline
Proline
Glycine
Threonine
Betaine
Glucose
Uracil
Uridine
Urocanate
Fumarate
Tyrosine
Histidine
Tryptophan
Phenylalanine
Adenine
Inosine
Adenosine
Formate
AMP
Nicotinate
Nicotinamide

Supplemental References

Dona AC, et al.. (2014). Precision High-Throughput Proton NMR Spectroscopy of Human Urine, Serum, and Plasma for Large-Scale Metabolic Phenotyping. *Analytical Chemistry*, 86(19):9887–9894.

Eddy SR. (1998). Profile hidden Markov models. *Bioinformatics*, 14(9):755–763.

Jensen PA, et al.. (2015). Miniaturized Plate Readers for Low-Cost, High-Throughput Phenotypic Screening. *Journal of Laboratory Automation*, 20(1):51–55.

Meyer F, et al.. (2008). The metagenomics RAST server—a public resource for the automatic phylogenetic and functional analysis of metagenomes. *BMC bioinformatics*, 9:386.

Powell S, et al.. (2014). eggNOG v4.0: nested orthology inference across 3686 organisms. *Nucleic Acids Research*, 42(D1):D231–D239.

R Core Team, 2013. R: A language and environment for statistical computing. Available at: http://www.r-project.org/.

Rho M, Tang H, Ye Y. (2010). FragGeneScan: predicting genes in short and error-prone reads. *Nucleic Acids Research*, 38(20):e191–e191.

Veselkov KA, et al.. (2009). Recursive Segment-Wise Peak Alignment of Biological 1 H NMR Spectra for Improved Metabolic Biomarker Recovery. *Analytical Chemistry*, 81(1):56–66.

Wang J, et al.. (2014). PNAS Plus: From the Cover: Dietary history contributes to enterotype-like clustering and functional metagenomic content in the intestinal microbiome of wild mice. *Proceedings of the National Academy of Sciences*, 111(26):E2703–E2710.
